# Identification and cause analysis on unplanned reoperations by text classification approach

**DOI:** 10.1038/s41598-025-22791-9

**Published:** 2025-11-10

**Authors:** Zhancheng Liang, Wenyang Huang, Hongyu Xu, Zhenkun He, ChunQiu Yuan, Yan Liang, Qiuquan Guo, Tianzhao Liu, Peipei Jia

**Affiliations:** 1 Shensi Lab, Shenzhen Institute for Advanced Study, UESTC, Shenzhen, China; 2 Wuyi University, Wuyishan, China; 3 Peking University Shenzhen Hospital, Shenzhen, China

**Keywords:** Unplanned reoperation, Medical quality control, Text classification, Medical text, Computer science, Information technology, Data mining, Data processing

## Abstract

Unplanned reoperations (URs) not only increase the hospitalization period and healthcare cost, but also raise the death risk of patients. The analysis of URs is thus significant for their quality control and reduction. However, the massive text data generated in hospitals makes the identification of URs a tedious task with potential bias. Current research on UR is limited to data analysis and lack automated classification using deep learning and natural language processing. Here we propose the UR-Net framework. It implements the UR identification and cause analysis by processing the long texts of ward round documentation and applying few-shot learning on multi-class cause classification. Our framework consists of the URNet-XL with a batch fusion method based on XLNet model, and the URNet-GT for cause classification based on the pre-trained model combined with feature extraction modules of multi-head attention and a bi-directional Gated Recurrent Unit. High weighted F1 scores of 96.34% and 93.37% are obtained for the respective processes in comparison with the baseline methods. The Area Under receiver operating characteristic Curve (AUC) of 97.86% indicates an excellent UR classification on the unbalanced dataset. Our approach provides a new route of UR identification and analysis with the potential of reducing its occurrence.

**Supplementary Information:**

The online version contains supplementary material available at 10.1038/s41598-025-22791-9.

## Introduction

An unplanned reoperation (UR) could occur to the patient after the initial operation due to various medical and nonmedical factors during the same hospitalization, as opposed to planned reoperation^1^. The medical quality control should be implemented if a patient returns to the operating room for an UR within 48 h or 31 days of a particular surgery. URs are significantly associated with elevated morbidity and mortality rates in postoperative patients. They not only prolong the average length of hospital stay, but also reduce the recovery rate of patients. Complications from the initial operation are defined as UR causes, which might be implicit in the doctor’s surgical records. Depending on these surgical records, the surgery can be identified as the planned or unplanned operation. If it was the latter, the reason has to be further analyzed to prevent more cases. The UR rate is the proportion of the number of URs in the total number of operations over a period. Hospitals implement rigorous medical quality control measures to ensure optimal patient outcomes and minimize complications. UR rate reflects the effectiveness of primary surgeries and postoperative care, and it has been used as a surgical quality index of health departments in numerous countries^2^. This metric has been widely recognized and applied in various healthcare systems to assess surgical outcomes and quality^3^. In 2022, the Nation Health Commission of China set an unprecedented goal of reducing the incidence of URs as one of the key goals for national healthcare quality improvement.

The reasons of URs are diverse, complicated and department-specific. Usually, it is due to issues with surgical technique^4^, anesthesia^5^ or infection control^6^, leading to negative postoperative consequences. For example, a study indicated that the patients of thoracic surgery would have URs mostly due to epidural hematoma, wound complications, implant complications, and inadequate decompression^7^. In a statistical analysis of URs for head and neck cancer, flap complications, infection, necrosis and bleeding were confirmed as the common causes of URs after head and neck cancer surgery^8^. Another study demonstrated the relationship between weight loss, tumor spread, prolonged operation time, decreased preoperative albumin and hematopoietic volume, and the incidence of complications and URs at the complex surgical site of the paracentral forehead flap^9^. The risk of UR was revealed to increase significantly when the first operation of patients with tertiary general surgery services was emergency surgery^10^. In addition, the correlation between UR and unplanned readmission was also discussed^11^.

At present, the analysis of URs mainly depends on the personal judgment of experienced doctors on surgical data. Such tasks consume a significant amount of the doctor’s time due to the massive data routinely generated in hospitals on a daily basis. Thus, to avoid potential bias in the tedious analysis, it is necessary to apply deep learning^12^ and natural language processing (NLP) in processing surgical data and making medical decisions. However, the early research on the identification and classification of UR only relied on the analysis of its statistical characteristics^1,13^. Such methods often simply analyze the rate of URs, and these factors can lead to UR and these factors can lead to UR relationship with the survival rate. For example, by using the CiteSpace knowledge graph^14^, the global concern about UR was disclosed to grow year by year. In the same way, most of the recent methods focus on univariate analysis, which are often performed through controlled studies or statistical tests. For instance, χ2 tests have been extensively used to analyze categorical data in various studies^15^. Univariate logistic regression has been applied in other works to assess the association between independent variables and outcomes^16^, with further refinements in specific applications^17^ and methodological extensions^18^. Additionally, multivariate analysis has gained prominence, including the use of logistic regression models to explore complex interactions^4,8^. Cox proportional risk models have also been widely utilized to investigate time-to-event data and hazard ratios^19^.

In 2023, Mason et al.^20^ analyzed surgeries from 700 hospitals using ACS-NSQIP data from 2012 to 2018. The study developed a prediction model for URs based on 29 non-laboratory preoperative variables using multivariable logistic regression for the first time. This study validated the accuracy of the model in predicting UR, and it highlights the importance for patient risk education and healthcare quality improvement. In 2024, Hassan et al.^21^ analyzed URs in 2,316 gender-affirming surgery patients using ACS-NSQIP data from 2012 to 2020. The study identifies key factors leading to URs by traditional machine learning methods. Recent research on URs and their causes focuses on traditional machine learning model validation, compassion and statistical analysis of data, but there is a lack of efficient URs and their causes classification using advanced deep learning and NLP methods to integrate the process of UR identification and cause analysis.

Currently, NLP technique has been widely applied in the research area of medical information. For instance, merging medical knowledge into pre-trained models was used to enhance their effect on downstream tasks^22^. Since medical records are often lengthy, event and entity extraction were employed to structure the text, with knowledge graphs incorporated to enhance the comprehension of medical information by models. This supports tasks such as Q&A and text summary generation, as demonstrated by Ramponi et al.^23^, Zhang et al.^24^, and Lai et al.^25^. Furthermore, medical entities from unstructured medical records were identified and analyzed to tailor the medical treatments, as discussed by Song et al.^26^, Yao et al.^27^, Zhang and Liang^28^, Li et al.^29^, Tang^30^, and Jia et al.^31^. Text classification tasks can also be performed to determine whether a patient is suffering from a particular disease or not, and to classify adverse event reports, as shown in the works of Barber et al.^32^, Mao et al.^33^, Lu et al.^34^, Ge et al.^35^, Yuan and Duan^36^, and Guo et al.^37^. Ultimately, these processes could assist in improving the healthcare quality.

Among others, incorporation of machine learning into medical text classification is currently attracting more research interest. For instance, a transfer learning for meta-learning and an ALBERT-based fusion Kalman-filter model were proposed for clinic text classification^38,39^. Preoperative prediction and injury identification also made use of the NLP technique^40^. In addition, the NLP was employed to evaluate the quality of end-of-life measures for cancer patients receiving palliative surgery^41^. Although the trained model has high sensitivity and accuracy, its performance is limited by specific keyword matching in the corpus. Moreover, the NLP was also used to identify postoperative complications to improve surgical quality^42^. Unlike previous methods, this approach not only pre-trained its model using a Chinese medical corpus, but also designed two different profiles to handle the long texts and the samples with very limited data separately. Incorporating these strategies into one framework could significantly enhance the prediction performance. Recently, a novel method for multi-dimensional health risk classification based on blood test data was proposed^43^. It combines feature elimination, self-feature weighting, and innovative feature selection techniques, which can process the massive and complex data generated in hospitals efficiently. Additionally, the RAPID framework for robust APT detection and investigation using context-aware deep learning demonstrates the framework’s ability to adapt to evolving data patterns and reduce false positives^44^.

Although the surgical data are complex including both structured data and unstructured data such as images and vital signs^45^, a ward round documentation (WRD) mainly comprises textual data, acting as the convergence point of surgical information. Based on the tertiary ward round system, a WRD comprises three paragraphs for the first (resident rounds), second (attending rounds) and third (chief rounds) ward round before the next surgery. It serves as a tool to assess a patient’s physiological and disease status between the primary surgery and the following surgery, thereby facilitating the UR determination. However, a WRD can often be so lengthy and complex that it takes doctors a significant amount of time to arrive at a conclusion. To expedite the manual decision-making process, doctors mark any critical sentence in the record that serves as evidence for a UR for consideration. The classification based on long text processing and attention mechanism^46^ can replace the manual identification to automatically extract key features. Furthermore, once an operation is identified as an UR, the cause needs to be found out, thereby preventing subsequent occurrences of that type and improving the quality of care ultimately. A quality control (QC) text records the diagnosis of the cause of a UR occurrence and summarizes them into a keyword for subsequent statistics. Thus, mining these data with language models in NLP can offer unique advantages and produce various meaningful results.

Since stringent quality control measures implemented by healthcare institutions, UR represents a small fraction of total surgical cases, and the number of QC texts is thus usually small. This rarity presents a unique challenge for data-driven approaches aimed at analyzing and improving surgical outcomes. Deep learning has revolutionized various fields, including medical informatics, by enabling the extraction of complex features from unstructured text data. However, traditional deep learning models often require large amounts of labeled data, and this process can be costly and time-consuming to acquire. To address this limitation, few-shot learning aims to learning from a small number of examples per class^47^. Pre-training has proven effective in improving model performance across a range of tasks by first learning generalizable features before fine-tuning on task-specific data, particularly with large-scale unsupervised data^48^. A real-world study by Fan et al.^70^ explored the influence of URs on hospitalized patients using the Diagnosis-Related Group (DRG) system. This study highlighted how URs are not only a significant clinical concern but also an economic one, impacting hospital resource allocation and patient discharge planning. It reinforces the need for predictive models that can help identify high-risk patients early and guide targeted interventions in surgical practice.

Recent advancements in medical AI validation have emphasized a critical shift from single-center retrospective studies toward multi-center and prospective validation as the gold standard for demonstrating clinical utility and generalizability^73^. Concurrently, there is growing emphasis on addressing model safety and bias, with frameworks emerging to quantify large language model (LLM) “hallucination” rates in medical text summarization^74^ and to mitigate algorithmic bias using synthetic data^75^. In the realm of Clinical Decision Support Systems (CDSS), integration is becoming deeper and more actionable, as next-generation AI-native systems are embedded into hospital information systems, delivering personalized recommendations with high physician acceptance rates^76^. These trends are further characterized by seamless integration with Electronic Health Records (EHRs), leveraging predictive analytics for personalized care, and utilizing Natural Language Processing (NLP) to extract insights from unstructured clinical notes^77^.

In alignment with these developments, we apply deep learning and NLP to UR classification to achieve its quantification in a model named UR-Net. UR-Net fundamentally advances surgical text analysis by overcoming critical limitations of conventional models like BioBERT^57^ and clinicalBERT^58^ in processing long medical narratives. Specifically, BERT-based architectures suffer from severe context truncation (512-token limit), which discards over 47.2% of causal relationships in WRD texts (e.g., average length of 801 tokens), such as multi-day complication sequences like “post-op day 7 infection → reoperation”. UR-Net is innovative because it combines batch fusion with an attention mechanism, allowing it to fully understand 1024-token clinical records. It helps preserve the continuity of cause-and-effect relationships over time, which regular BERT-based models often break up. Moreover, we propose an integrated framework to streamline the pipeline for unplanned reoperation identification and cause extraction, simplifying the manual decision-making process while ensuring accurate identification. This model has the potential to aid in improving surgical safety and quality control of URs in hospitals.

To summarize, our contributions are as follows.

- We developed a deep learning framework of the UR-Net to identify URs and extract their causes from WRD and QC data. Our UR-Net consists of the URNet-XL with a batch fusion method based on XLNet^49^ model, and the URNet-GT for cause classification based on NEZHA (NEural ContextualiZed Representation for CHinese LAnguage Understanding)^50^ pre-trained model combined with multi-head attention and Bidirectional Gated Recurrent Unit (BiGRU)^52^.
- We applied a stratified batch sampler to ensure an equal ratio of case and control samples in each batch, and employed a focal loss to address the class imbalance. Batch fusion is used for processing the long texts in UR identification, while the fast gradient method (FGM)^51^ is exploited for adversarial training to enhance the robustness of few-shot learning. Moreover, five-fold cross-validation addresses the issue of small training dataset in the cause extraction.
- We validated our UR-Net for UR identification and cause extraction on two datasets, showing the efficiency and quality of deep learning methods in improving the UR analysis.

## Materials and methods

**Fig. 1:**
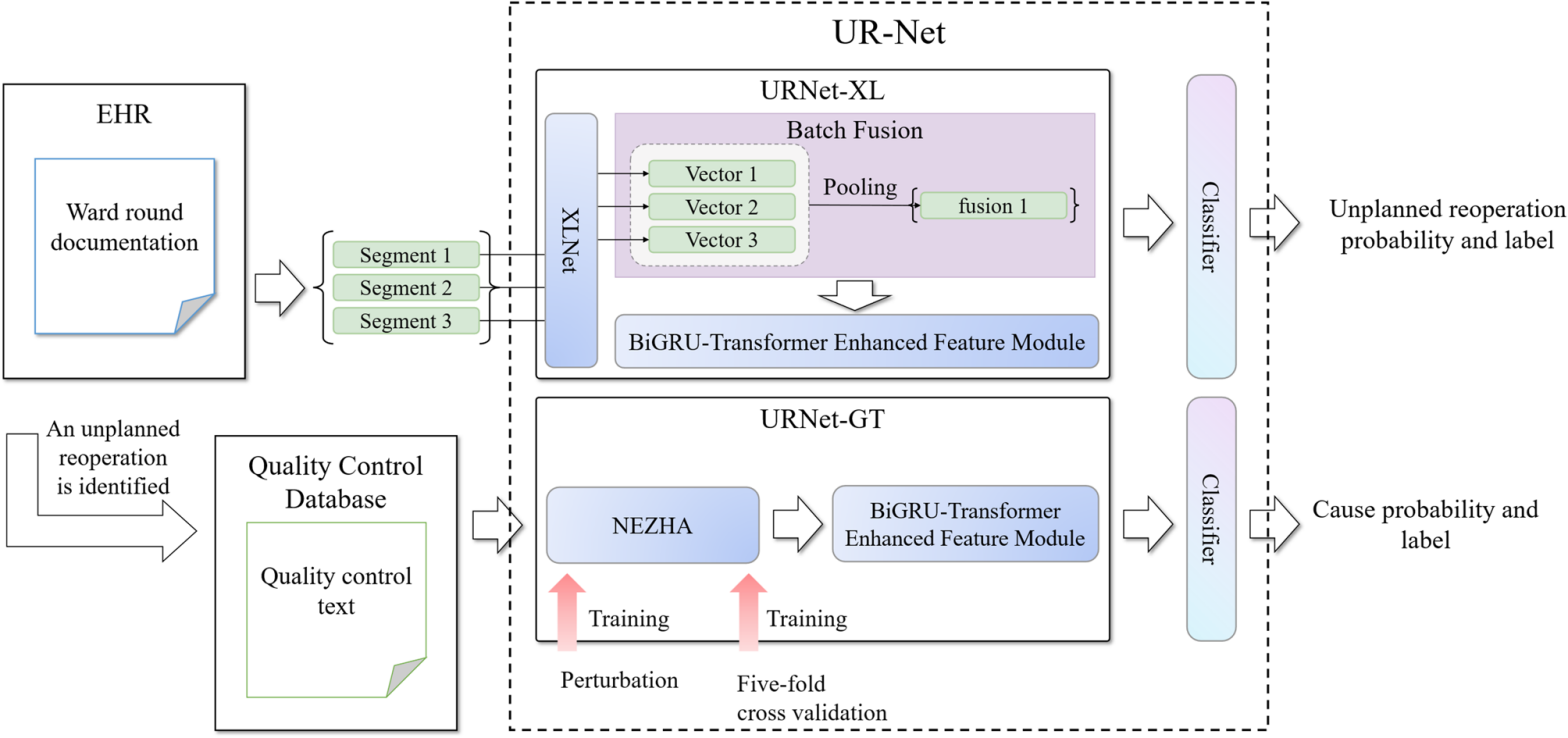
Architecture of the UR-Net framework for unplanned reoperation (UR) identification and cause analysis. The system operates sequentially: (1) Ward round documents from EHR systems are first processed by the URNet-XL sub-network. This module employs XLNet encoding, batch fusion, pooling, and a BiGRU-Transformer enhanced feature module (GT-EFM, including a BiGRU and a transformer encoder) to predict the probability and label of a UR. (2) Only if a UR is identified, the framework proceeds to the next stage. (3) Quality control (QC) texts from the QC database are then processed by the URNet-GT sub-network, which utilizes NEZHA encoding, an GT-EFM, and incorporates five-fold cross-validation during training for robust cause classification, ultimately outputting the cause probability and label.

The data for current research is obtained from Peking University Shenzhen Hospital cover the period from 2015 to 2021. Specifically, WRD data is extracted from electronic health record (EHR) database, and the QC data is extracted from UR quality control database. All the data in this study were anonymized and de-identified prior to analysis to protect the participants’ confidentiality. Due to the retrospective nature of the study, the Ethics Committee of the University of Electronic Science and Technology of China waived the need for obtaining informed consent. The study approved by the Ethics Committee of the University of Electronic Science and Technology of China. The study conducted in full compliance with the principles in the Declaration of Helsinki. The framework of UR-Net has two main parts as shown in Fig. 1:

1. URNet-XL for UR identification. The structure of model “URNet-XL” is optimized for processing long texts of ward round records. We choose XLNet as the pre-training model. The subsequent data is then fed into a linear layer for batch fusion and a feature extraction module to obtain the classification result. To achieve the judgement of the surgical category by surgical procedures, we utilize comprehensive approaches such as stratified sampling, linear warmup, and gradient cropping.
2. URNet-GT for cause extraction. This model pertains to a small sample classification task. We mainly use the NEZHA pre-training model and optimize the fine-tuning with the FGM. Manually tagged keywords are spliced onto the QC texts, where the maximum text length is no longer than 512 characters. These texts are encoded by NEZHA pre-training model and later processed by BiGRU and transformer encoder for further feature extraction^53^. The output is then fed into a linear layer for final classification. We integrate hierarchical sampling and linear warmup strategy to extract the reasons of URs.

### Long text processing for unplanned reoperation identification

#### Data preprocessing

Surgery data and WRDs are exported from the EHR. These two datasets are merged using case number, admission/discharge dates, and surgery start time as keys. Specifically, surgeries for the same patient during a single hospitalization are sorted chronologically, and their corresponding WRDs are extracted to form the training text corpus. The final dataset includes records of surgery types (labels) and their associated WRDs, with a total volume of 2,876 entries. The surgeries are categorized into 6 types: first surgery, UR, planned reoperation, canceled surgery, therapeutic surgery, and diagnostic surgery.

**Fig. 2:**
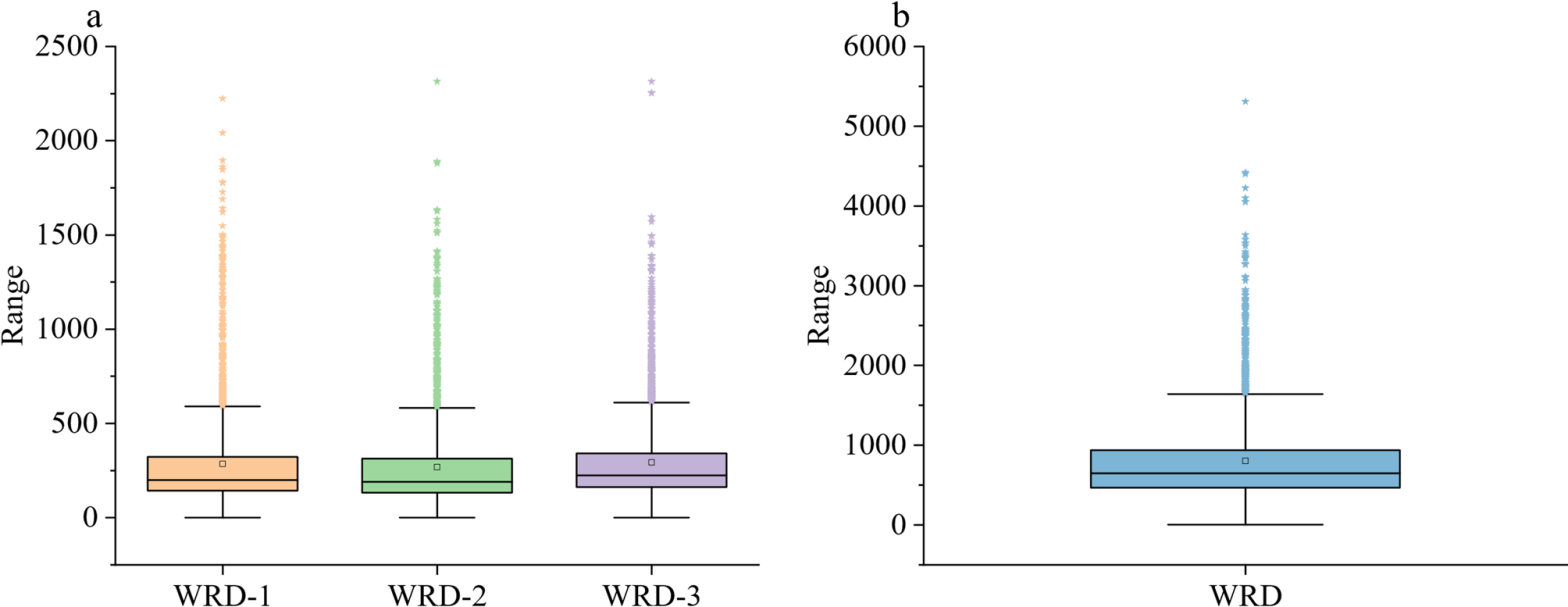
Length distributions of paragraphs in ward round records (**a**) and the concatenated texts (**b**). In the boxplots, the line shows the median, the box indicates the interquartile range (IQR), and the whiskers stretch to 1.5 times the IQR. The maximum sequence lengths of three parts can all be over 2,000 characters. The average lengths of three parts are 285, 269 and 292 characters, respectively. The ranges within 1.5 times the IQR of three parts are less than 1000 characters, while the ranges of each part of WRD are less than 600 characters. 75% of concatenated WRD texts of three parts are less than 1,000 characters, but the medium and the average lengths reach 648 and 801 characters, respectively.

To streamline the task, we first exclude first surgeries (as they are non-UR cases) to reduce model interference. We then group all non-UR, non-emergent surgeries (including planned reoperations, therapeutic, and diagnostic surgeries) into a unified “planned reoperation” category. This transforms the task into a binary classification problem: identifying UR events among all reoperations. After filtering out ineligible records (e.g., incomplete or non-reoperation cases), 2,514 WRDs remain, comprising 1,847 planned reoperations and 667 URs. Notably, the length of these records often exceeds 2,000 characters (as shown in Fig. 2), which constrains model selection—an issue we address in the modeling section.

To ensure the reliability of ground truth labels, three senior clinicians (2 from Gastroenterology, 1 from Critical Care Medicine, each with $\:\ge\:$10 years of experience) independently annotated the dataset using a standardized guideline. This guideline explicitly defined UR events (e.g., URs due to unforeseen complications) and 12 cause categories. Inter-annotator agreement was quantified via Cohen’s Kappa coefficient:

- For UR identification (binary: UR vs. non-UR), $\:\kappa\:$=0.81(95% CI: 0.72–0.89), indicating “almost perfect agreement”^71^.
- For UR cause classification (12 categories), $\:\kappa\:$=0.75(95% CI: 0.65–0.85), reflecting “substantial agreement.”

In clinical practice, emergent URs interrupt the standard WRD cycle, resulting in incomplete records prior to surgical intervention. To address this data incompleteness in model input processing, ward-round notes were structured as a concatenated sequence: 〈First_Round〉< sep>〈Second_Round〉< sep>〈Third_Round〉, where < sep > functions as a domain-specific separator token. During dataset construction, instances with missing rounds underwent adaptive preprocessing: redundant < sep > tokens corresponding to absent records were systematically eliminated. This approach preserves the sequential integrity of available documentation while minimizing artifactual noise from null values.

#### URNet-XL architecture

##### Pre-trained model

Several encoders in language models can improve the ability to capture long-range dependencies. Although Long Short-Term Memory (LSTM) uses gating and gradient clipping^54^, its ability to capture long-range dependencies is limited, typically to about 200 tokens^55^. Transformer^46^ enables the direct linkage between words via a self-attention mechanism, enhancing its ability to capture long-range dependencies in text, but it still has a fixed context length. Transformer-XL (eXtra Long)^56^ employs the segment-level recurrence with state reuse and relative positional encodings methods to establish textual long-term dependencies and solve the problem of contextual fragmentation. The BERT^48^ model performs well in classification of texts with less than 512 tokens, not sufficient for our case of WRDs. BioBERT and ClinicalBERT are designed for biomedical and clinical text, respectively. BioBERT fine-tunes BERT using large-scale biomedical corpora. ClinicalBERT adapts BioBERT specifically for clinical text by fine-tuning on clinical notes and medical records, improving its performance on clinical text classification tasks. However, these models are still constrained by BERT’s fixed-length input of 512 tokens. Models like Longformer^59^, ERNIE 3.0^60^ and XLNet are designed for long text processing. Longformer uses a sliding window mechanism to handle long documents efficiently, but its localized attention could miss important details, especially in complex medical texts. ERNIE 3.0 employs a multi-stream mechanism for long text, though it may struggle with modeling long-range dependencies compared to XLNet’s autoregressive approach. Although XLNet has higher attention computation complexity compared to Longformer and ERNIE 3.0, its permutation-based pretraining method excels at processing complex semantics in the long text. This capability is particularly beneficial for medical text analysis. Therefore, XLNet is more adapted to our task in processing medical long texts.

##### BiGRU-transformer enhanced feature module

**Fig. 3:**
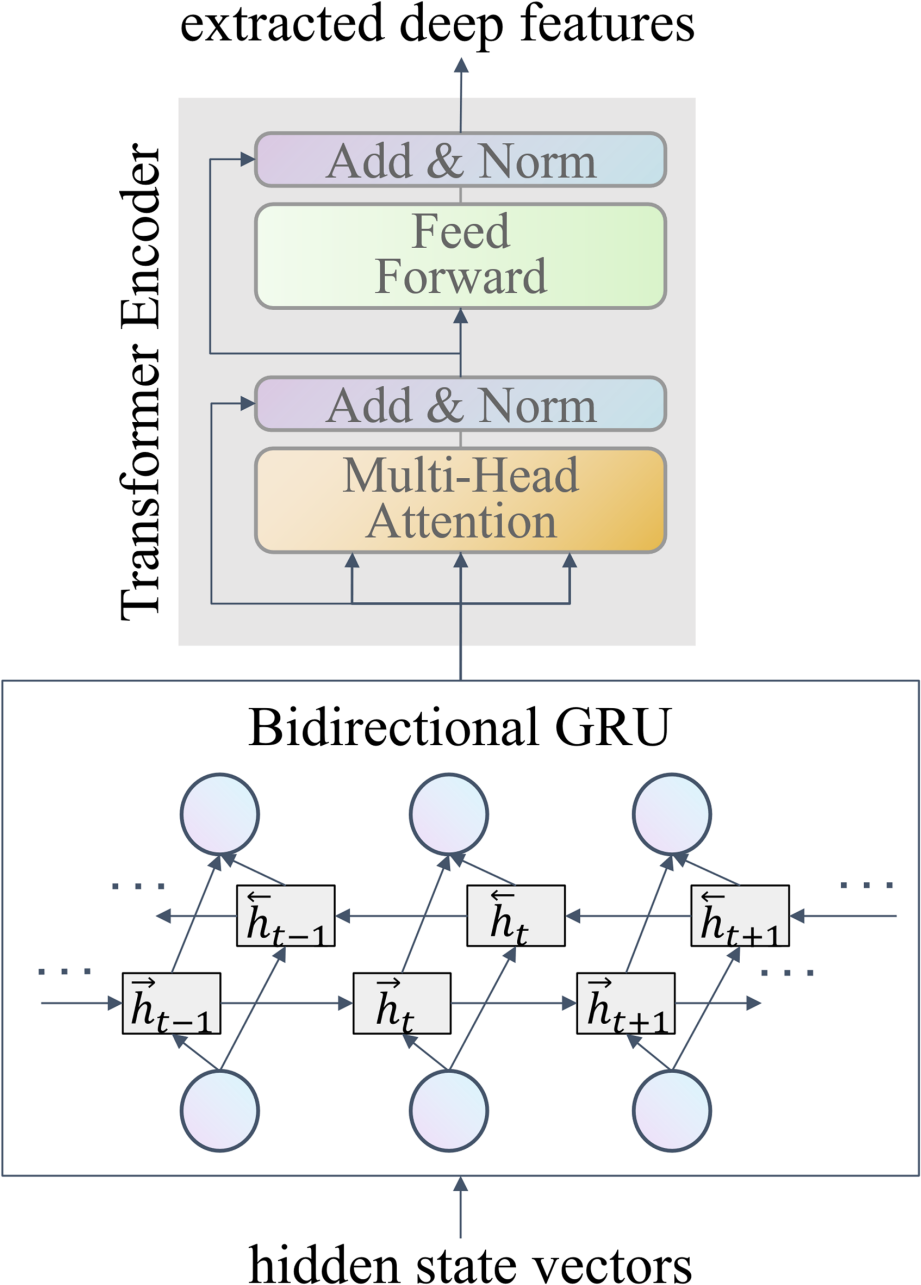
Further feature extraction module. The module consists of a BiGRU and a transformer encoder. It takes the output vectors from the pre-trained model as input and processes them to capture complex, high-level representations of the text. The BiGRU component helps in capturing sequential dependencies by processing the text in both forward and backward directions ($\:{h}_{i}^{t}=\left[\overrightarrow{{h}_{i}^{t}};\overleftarrow{{h}_{i}^{t}}\right]$), while transformer encoder enhances the model’s ability to focus on relevant parts of the input text through self-attention mechanisms. The result is a set of refined, high-dimensional features that represent the meaning of the text for further processing.

XLNet learns complex language patterns. However, when working with long medical texts, not all parts of the text are equally important for understanding. Traditional text classification methods rely on a special token, called the [CLS] token, to represent the entire sentence or document. In our work, we improve the process of extracting important information from medical records by introducing the ​BiGRU-Transformer Enhanced Feature Module​ (GT-EFM), and it consists of a BiGRU and a transformer encoder (shown in Fig. 3). The BiGRU is a more efficient version of the LSTM model. It simplifies the structure by reducing the number of parameters, while still keeping the ability to process information well. Moreover, BiGRU extracts features from the hidden layers in both forward and backward directions of the text, effectively enhancing the model’s ability to capture contextual information. Additionally, BiGRU increases the long-distance dependency length of the transformer, preventing overfitting while improving stability. Generally, doctors determine URs by examining certain elements of texts in the typically long medical records. We use an attention mechanism to mimic this human ability to focus on key information. After the BiGRU, the attention mechanism helps the model highlight the most relevant details, which makes it easier for the model to understand the overall meaning. By combining this attention mechanism with medical text data, our model can better capture important medical terms and improve its ability to identify the type of surgery, leading to more accurate results.

For a given sentence, we represented each word and character by utilizing the pre-trained word embeddings from XLNet. The word embeddings and the initial extracted features will be fed to a BiGRU layer. The hidden state of BiGRU can be expressed as follows:

1 $$\:\overrightarrow{{h}_{i}^{t}}=\overrightarrow{GRU}\left({x}_{i}^{t},{\overrightarrow{h}}_{i-1}^{t}\right)$$

2 $$\:\overleftarrow{{h}_{i}^{t}}=\overleftarrow{GRU}\left({x}_{i}^{t},{\overleftarrow{h}}_{i+1}^{t}\right)$$

3 $$\:{h}_{i}^{t}=\left[\overrightarrow{{h}_{i}^{t}};\overleftarrow{{h}_{i}^{t}}\right]$$

where $\:{x}_{i}^{t}$ is the token representation,$\:\:\overrightarrow{{h}_{i}^{t}}$ and $\:\overleftarrow{{h}_{i}^{t}}$ denote the $\:t$-th forward and backward hidden state of GRU layer, and $\:\left[\cdot\:\right]$ represents the concatenation operation.

The transformer employs $\:h$ attention heads to implement self-attention on an input sequence separately, and a multi-head attention integrates the output of each attention head. Given a sequence of vectors $\:X$, a query vector $\:Q$ is used to selectively retrieve relevant information with attention:

4 $$\:Att\left(Q,\:K,V\right)=\:softmax\left(\frac{Q{K}^{\top\:}}{\sqrt{{d}_{k}}}\right)\cdot\:V$$

5 $$\:K=X{W}^{K},V=X{W}^{V}$$

where $\:{W}^{K}$ and $\:{W}^{V}$ are learnable parameters. Then multi-head attention can be defined as Eq. (6) ~ (7):

6 $$MulAtt = \left( {z_{1} \oplus z_{2} \oplus \cdots \oplus z_{h} } \right) \cdot W^{O}$$

7 $$\:{\mathcal{z}}_{i}=Att\left(Q{W}_{i}^{Q},K{W}_{i}^{K},V{W}_{i}^{V}\right)$$

where $\:\oplus\:$ denotes concatenation, and $\:{W}^{O}$, $\:{W}_{i}^{Q}$, $\:{W}_{i}^{K}$, $\:{W}_{i}^{V}$ are learnable parameters.

##### Processing long medical text with URNet-XL

**Fig. 4:**
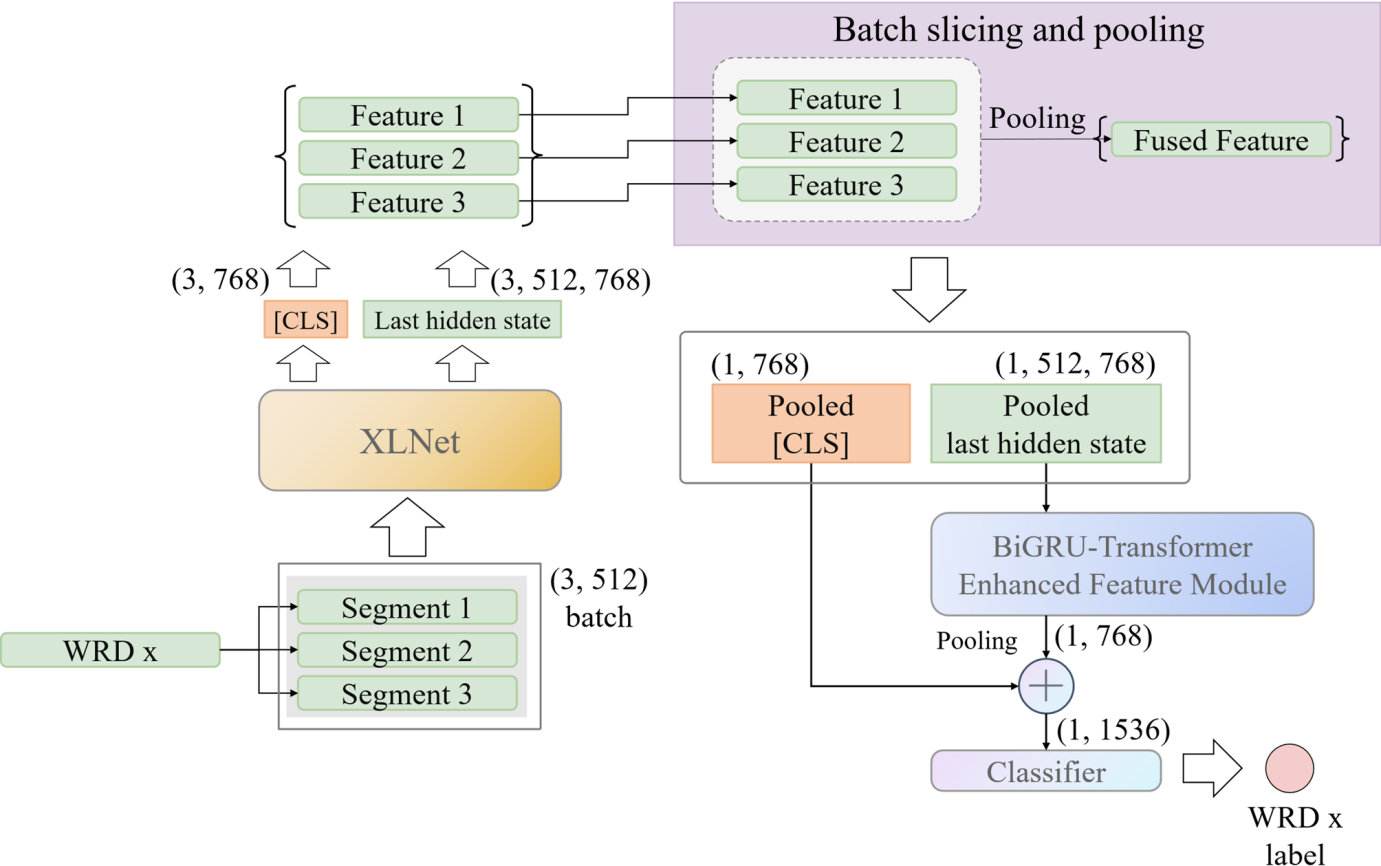
URNet-XL: Framework of long text processing model. Input ward round documents (WRDs) are batched into groups of three (Segment 1, Segment 2, Segment 3; max sequence length: 512 tokens). The ​Batch Slicing and Pooling​ module processes each WRD ​independently​ through XLNet, generating three encoder outputs ((3, 768) vectors). Each output undergoes pooling to extract its last hidden state ((1, 768) per WRD). These pooled states are fused and forwarded to the ​BiGRU-Transformer Enhanced Feature Module​ (GT-EFM)​​ for refined feature learning. Simultaneously, the pooled [CLS] embedding ((1, 768)) – representing global context – is concatenated with the GT-EFM output ((1, 768)), forming a fused feature vector ((1, 1536)). This vector is classified to predict UR occurrence (label). (Model hidden size: 768).

Our approach is schematically shown in Fig. 4. For a single WRD text $\:X$ (comprising three clinically structured segments: resident round record $\:{S}_{1}$, attending round record $\:{S}_{2}$, and chief round record $\:{S}_{3}$), each segment $\:{S}_{i}$ (where $\:i\:=\:1,\:2,\:3$) is first truncated to $\:\le\:512$ tokens to match the input constraint of the XLNet-base model:

8 $$\:X=\left({S}_{1},{S}_{2},{S}_{3}\right)$$

After encoding by XLNet, each segment outputs two key feature vectors: The [CLS] token embedding $\:{H}_{0}\in\:{\mathbb{R}}^{3\times\:768}$, which encapsulates the global semantic information of segment $\:X$; The final-layer hidden state matrix $\:{H}_{l}\in\:{\mathbb{R}}^{3\times\:512\times\:768}$, which retains fine-grained local features (e.g., clinical terms like “postoperative infection” or “surgical site bleeding”):

9 $$\:{H}_{0},{H}_{l}=XLNet\left(X\right)$$

where $\:{\text{H}}_{0}=\left({h}_{1}^{0},{h}_{2}^{0},{h}_{3}^{0}\right)$ signifies the triplet of [CLS] token embeddings output by the pretrained model, and $\:{H}_{l}=\left({h}_{1}^{l},{h}_{2}^{l},{h}_{3}^{l}\right)$ denotes the triplet of hidden state vectors corresponding to the [CLS] tokens, obtained at the very last layer of the pretrained model before the final output is produced.

To integrate semantic information across segments without losing cross-segment causal relationships (e.g., “infection confirmed in $\:{x}_{2}\to\:$ reoperation planned in $\:{x}_{3}$”), one batch of the data is processed: three encoded texts in one batch are taken out at a time, and the result of this batch outputs. The pooled last hidden state vector consolidates the rich semantic features extracted by the model. The process is defined as Eqs. (10) and (11):

10 $$\:{h}_{0}=BF\left({H}_{0}\right)$$

11 $$\:{h}_{l}=BF\left({H}_{l}\right)$$

where $\:BF\left(\cdot\:\right)$ represents the batch fusion method, $\:{h}_{0}$ denotes the pooled [CLS] token embedding obtained from batch fusion, and $\:{h}_{l}$ is the pooled last hidden state vector obtained from batch fusion.

One third of the original batch of encoder outputs enters further feature extraction module and an average pooling layer in turn. The average pooling operation serves to feature down-sampling. The operation reduces the feature size while maintaining important features and prevents overfitting. By contrast, max pooling selects the most “salient” segment $h_{i}^{*} = \arg \mathop {\max }\limits_{i} \parallel h_{i} \parallel$, which risks ignoring subtle but clinically important interactions between moderate segments (e.g., gradual changes in vital signs). While attention weights can dynamically emphasize relevant segments, they introduce additional computational complexity and overfit to noise in small datasets.

12 $$\:{h}_{l}^{{\prime\:}}=AvgPool\left(FFEM\left({h}_{l}\right)\right)$$

where $\:FFEM\left(\cdot\:\right)$ represents the further feature extraction module, and $\:AvgPool\left(\cdot\:\right)$ denotes the average pooling operation.

The pooled output $\:{h}_{l}^{{\prime\:}}$is concatenated with the CLS Embeddings of the same size (i.e. one third of the original batch size) after batch fusion. The linked results pass through a linear layer for classification, and the model finally outputs the category of the operation $\:\widehat{y}$:

13 $$\:p=softmax\left({h}_{0}\oplus\:{h}_{l}^{{\prime\:}}\right)$$

14 $$\:\widehat{y}={arg}\underset{i}{{max}}{p}_{i}$$

where $\:p$ is the computational probability, $\:{p}_{i}$ denotes the probability of the $\:i$-th category, $\:{h}_{0}\oplus\:{h}_{l}^{{\prime\:}}$ denotes the concatenation of $\:{h}_{0}$ and $\:{h}_{l}^{{\prime\:}}$, and $\:\widehat{y}$ is the predicted label of the model.

A dropout layer then invalidates certain neurons’ outputs in order with a specific probability to improve the generalizability of the model. Additionally, the focal loss^61^ helps with the sample imbalance issue to some extent. As the network depth increases, gradient vanishing or gradient explosion occurs during model training. We used a residual connection^62^ to simplify the learning process, enhance the gradient propagation and increase the generalization capability of our model at the same time.

Unlike arbitrary text segmentation (e.g., sliding windows), batch fusion method segments WRDs based on the tertiary ward round system (a standard clinical workflow in Chinese hospitals), ensuring that critical clinical decisions are not split across segments, and hierarchical clinical information of WRD is retained in its natural sequence. This segmentation logic aligns with how clinicians manually review WRDs to determine URs, minimizing artificial semantic fragmentation and enhancing the clinical interpretability of the fused features.

### Few-shot learning for cause classification

#### Data preprocessing

We collected 820 diagnoses from the QC database for the URs. Each of the diagnosis texts contain 80 to 420 characters. Twelve causes and their corresponding number of URs are shown in Table 1. “Bleeding” results in the most URs, while “thrombus” and “implant loosening” are rare. Thus, the task turned to a few-shot learning with multiclass classification on UR causes, requiring the model to classify multiple categories of UR causes with only a small amount of data available for each category.

**Table 1:** Statistics on the causes for URs.

Cause	(*n* = 820)	(% of total)
Bleeding	352	42.93
Leakage, fistula, obstruction	103	12.56
Infection	87	10.61
Not achieve the desired effect	70	8.54
Flap crisis	46	5.61
Splitting or poor healing of incision	45	5.49
Implant loosening	30	2.93
Cerebral edema, cerebral embolism	24	2.66
Pathology	18	2.20
Thrombus	15	1.83
Subsidiary injury of an operation	15	1.83
Foreign body legacy	15	1.83

The table presents the distribution of causes for unplanned reoperations (URs) in a cohort of 820 cases. The causes are listed alongside the number of occurrences and the percentage they represent of the total number of cases. The most common cause of URs was bleeding, accounting for 42.93% of cases, followed by leakage, fistula, and obstruction at 12.56%. Other causes include infection, failure to achieve the desired effect, flap crisis, and issues related to incision healing, among others.

We united tags of the same meaning (e.g. “flap crisis” and “flap vascular crisis” to “flap crisis”) to standardize the experimental setup. To enable knowledge learning with high performance, we manually annotated the diagnostic text with its keywords. Since the diagnosis texts of different departments have their own characteristics, we concatenated their department names and keywords with the corresponding diagnosis text through “[SEP]” (a special separator that is recognized by pre-trained model). Besides, certain special characters were removed such as quotation marks and excess spaces. Finally, to enable the model to understand the diverse knowledge from different departments, we also concatenated the diagnosis text with the department annotation through “[SEP]”. The final input text $\:x$ is constructed according to Eq. (15):

15 $$\:x=q\oplus\:k\oplus\:d$$

where $\:\oplus\:$ represents the concatenation operation with [SEP], $\:q$ is the diagnosis text, $\:k$ is the keyword, and $\:d$ denotes the department.

#### URNet-GT architecture

**Fig. 5:**
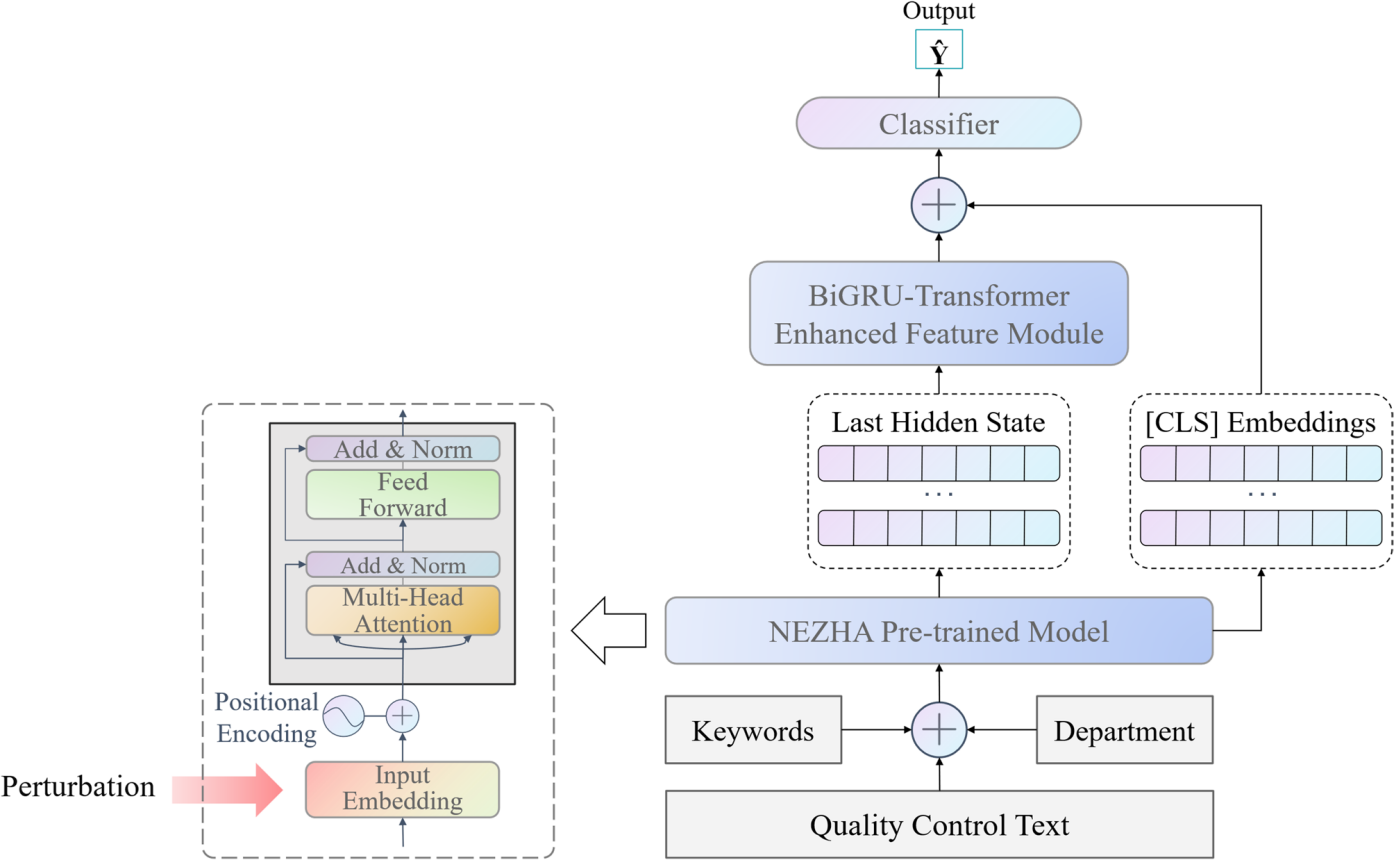
URNet-GT: UR cause classification architecture. The model processes three input streams: (1) Quality Control (QC) text describing the UR case, (2) manually annotated keywords, and (3) corresponding department identifiers. Input Embeddings are perturbed via Fast Gradient Method (FGM) to enhance robustness against adversarial examples. The NEZHA pre-trained model processes the combined input, incorporating Positional Encoding and Multi-Head Attention. Its final hidden state undergoes further transformation in the BiGRU-Transformer Enhanced Feature Module​ (GT-EFM), comprising a Bidirectional Gated Recurrent Unit (BiGRU) and a Transformer Encoder layer. Classification leverages the pooled representation of the [CLS] token (representing global context) fused with the refined features from the GT-EFM.

In URNet-GT (shown in Fig. 5), we chose NEZHA as our pre-trained model. Its relative position encoding that calculates automatically can learn the relationship between characters better than the absolute position encoding of BERT. In addition, the Whole Word Masking (WWM) strategy demonstrates improved efficacy in Chinese sentences composed of several words compared to single-word sentences. Note that certain special words may significantly mislead the final classification result. For example, the presence of the word “bleeding” will lead the model to attribute the cause to “bleeding” with high probability. GRU outperforms RNN in both information preservation and convergence due to its special structure^63^. Therefore, BiGRU was implemented to effectively integrate contextual information, leading to an enhanced semantics understanding.

Our analysis of diagnoses disclosed that not the entire text in the QC database described the causes of URs. Accordingly, professionals usually pay more attention to certain key phrases during the cause determination. An attention mechanism is thus used to simulate this characteristic as well. It can score each dimension of the input, and then the features are weighted according to their scores to highlight the impact of important features on downstream models or modules. With the help of multi-head attention mechanism, our model can concentrate on the important parts of a text. The process of UR cause classification is as Eq. (16) ~ (19):

16 $$\:{h}_{0},{h}_{l}=NEZHA\left(x\right)$$

17 $$\:{h}_{l}^{{\prime\:}}=AvgPool\left(FFEM\left({h}_{l}\right)\right)$$

18 $$\:p=softmax\left({h}_{0}\oplus\:{h}_{l}^{{\prime\:}}\right)$$

19 $$\:\widehat{y}={arg}\underset{i}{{max}}{p}_{i}$$

where $\:{h}_{0}$ denotes [CLS] token embedding, $\:{h}_{l}$ is the last hidden state vector obtained from the pretrained model, $\:FFEM\left(\cdot\:\right)$ is the further feature extraction module, $\:AvgPool(\cdot\:)$ represents the average pooling operation, $\:\oplus\:$ denotes the concatenation, and $\:{p}_{i}$ is the probability of the $\:i$-th category.

##### Loss function

The distribution of UR causes in our dataset is highly imbalanced, with certain classes having significantly more samples than others. As a result, the loss function is predominantly influenced by the majority classes. To mitigate this issue, focal loss is employed, which assigns higher weights to harder-to-classify samples, thereby encouraging the model to focus more on minority and challenging cases. It is defined as follows:

20 $$\:FL\left({p}_{t}\right)=-{\alpha\:}_{t}{\left(1-{p}_{t}\right)}^{\gamma\:}\text{log}\left({p}_{t}\right)$$

where $\:{p}_{t}$ is the probability of ground truth in the softmax output distribution. The hyperparameters $\:\alpha\:$ and $\:\gamma\:$ are employed to balance the loss. The focal loss can be intuitively understood as the multiplication of cross entropy of $\:-\text{log}\left({p}_{t}\right)$ by $\:{\alpha\:}_{t}{\left(1-{p}_{t}\right)}^{\gamma\:}$. Focal loss primarily reduces the loss for samples that are classified correctly while maintaining the loss for misclassified samples, thereby increasing the weight of misclassified samples in the loss function. Meanwhile, $\:{p}_{t}$ also represents the difficulty level of classification, with $\:{p}_{t}$ being directly proportional to classification confidence. As $\:{p}_{t}$ approaches to 1, the sample is easier to classify, and the focal loss decreases. Conversely, the smaller the $\:{p}_{t}$, the harder the classification task. Therefore, focal loss effectively increases the weight of the difficult-to-classify samples in the loss function, thereby enhancing the accuracy of the model while giving more emphasis to hard-to-classify samples.

##### Adversarial training by FGM

FGM is a training technique developed to enhance model robustness by introducing carefully crafted perturbations during the training process. It enables the model to cope with challenging conditions, particularly in scenarios characterized by limited data availability, thereby improving its performance in unfamiliar or difficult situations. As an adversarial training method, FGM can improve our model’s robustness in few-shot learning. It assumes the embedding representation of the input text sequence $\left[ {v_{1} ,v_{2} , \ldots ,v_{T} } \right]$ as $\:x$. Then the small perturbation is applied each time as:

21 $$r_{{adv}} = \in \cdot g/\parallel g\parallel _{2}$$

22 $$\:g={\nabla\:}_{x}L\left(\theta\:,x,y\right)$$

where $\in$ is the constraint of perturbation, $\:g$ is the gradient of the loss function, and $\:L2$ regularization is carried out on the gradient. FGM moves the input one step further in the direction of loss ascent, causing the model loss to ascend in the fastest direction, thereby generating an attack. Therefore, our model can identify more robust parameters in the optimization phase to deal with the attacks against the samples and avoid overfitting in our small sample dataset. FGM is not inherently designed to expedite the convergence of a model. It generates adversarial examples for use in adversarial training, thereby enhancing the robustness of the model. However, through the process of adversarial training, FGM can incidentally influence the convergence process of the model.

##### Five-fold cross-validation

Cross-validation is a robust statistical technique for evaluating model performance, particularly with limited data. In five-fold cross-validation, the dataset is divided into five equal subsets. The model is trained on four subsets and validated on the fifth, rotating this process across all subsets. This technique ensures each data point is used for both training and validation, optimizing data utilization and enhancing the model’s learning capacity. Cross-validation provides a reliable performance assessment by averaging results across multiple folds, ensuring generalization to new, unseen data.

Overall, in the output part of the pre-training model, a BiGRU layer and a multi-head attention layer are added after the last hidden state of the model. The attention layer extracts the keywords in the sentences, and BiGRU improves the memory ability of the model to achieve more accurate predictions. The outputs are concatenated with those outputs of CLS embedding for classification, dropout for preventing overfitting and focal loss for alleviating label imbalance problem, respectively.

## Experiments and results

### Unplanned reoperation identification

#### Experimental setups

We took the “Chinese-XlNet-base” model with 12 layers and 768 of hidden size as our pre-trained language model and the focal loss as our loss function. The initial learning rate for BertAdam was set to be 2e-5. We chose “warmup linear” as our warmup schedule with the warmup proportion of 0.1. The maximum sequence length of token was 512 and batch size was 24. 15 epochs per fold were set in five-fold cross-validation training. In addition, an “early stop” strategy was implemented on each epoch to save training time. If the F1 score lasting three epochs cannot be improved, the training will terminate early. We used stratified sampling on the dataset to create the training set, validation set and testing set in a ratio of 8:1:1 (shown in Supplementary Table S1). We evaluated the performance of our model on the testing dataset by Precision (P), Recall (R), F1 score and the Area Under receiver operating characteristic Curve (AUC). Their formulas are as follows:

23 $$\:P=\frac{TP}{TP+FP}$$

24 $$\:TPR=R=\frac{TP}{TP+FN}$$

25 $$\:F1=\frac{2\times\:P\times\:R}{P+R}$$

26 $$\:FPR=\frac{FP}{FP+TN}$$

where TP, FP, TN, FN stand for true positive, false positive, true negative and false negative numbers of case in the UR prediction, respectively. The Receiver Operating Characteristic (ROC) curve is a graphical representation of the performance of a binary classification model. The AUC of ROC measures the ability of a classifier to correctly rank positive samples higher than negative ones^64^. It is calculated by the area under the curve of true positive rate (TPR) against the false positive rate (FPR). Usually, the AUC is not sensitive to sample equalization, allowing for a large gap between the actual positive and negative. The high AUC value indicates the high classifier performance.

We compared our model with four baseline models in the UR identification to validate its performance and generalization. As a WRD text classification model, it is first compared with BERT (a representative text classification model in NLP). URNet-XL is also compared with several state-of-the-art models in the medical text classification domain, including BioBERT and Clinical BERT. In addition, considering our long text data, ERNIE 3.0, Longformer and XLNet capable of long text processing are included in our comparison. These models represent the most advanced approaches for long text classification tasks and have been widely applied in various domains. Moreover, we performed the ablation experiment to verify the effectiveness of the modules in URNet-XL.

#### Results

The results of different methods under the same hyperparameters of the batch fusion are shown in Table 2. In comparison with the baseline models, our URNet-XL demonstrates the best performance on all metrics. Particularly, the highest recall of 95.83% reflects its excellent ability to identify true URs, while the highest AUC of 97.86% indicates superior discrimination between positive and negative URs. The standard deviations in URNet-XL are lower than those of the other models, indicating consistent performance across different data splits. This suggests that URNet-XL is not only the top performer but also the most stable model. In contrast, models like BERT and BioBERT show higher standard deviations, reflecting greater variability in their results. While BERT performs well in terms of precision, its recall and AUC are lower than those of URNet-XL, indicating less robust identification of true URs and poorer classification performance. The larger standard deviations in Longformer highlight its sensitivity to data variations, with more fluctuating results across folds.

**Table 2:** Results of methods with batch fusion.

Method	*P* (%)	*R* (%)	F1 (%)	AUC (%)
BERT	90.00 ± 0.89	91.68 ± 0.92	90.83 ± 0.81	93.92 ± 0.88
BioBERT	89.12 ± 0.85	91.45 ± 0.89	90.27 ± 0.87	92.67 ± 0.89
ClinicalBERT	90.25 ± 0.82	92.10 ± 0.83	91.16 ± 0.80	93.10 ± 0.85
ERNIE3.0	87.00 ± 0.95	90.63 ± 0.93	88.78 ± 0.89	92.50 ± 0.91
Longformer	83.33 ± 0.78	93.75 ± 0.73	88.24 ± 0.75	92.98 ± 0.79
XLNet	88.35 ± 0.85	94.79 ± 0.81	91.46 ± 0.84	94.80 ± 0.87
URNet-XL (ours)	**96.84 ± 0.84**	**95.83 ± 0.76**	**96.34 ± 0.82**	**97.86 ± 0.71**

Bold numbers indicate the best result in each column. The values are presented as average value ± standard deviation (Std) from the five-fold cross-validation

With the same hyperparameters and pre-trained model, we further compared the results of different method combinations, shown in Supplementary Table S2. In conclude, ‘GRU + TF’ obtains the best performance. Based on ‘XLNet’, the transformer encoder module in ‘XLNet-TF(RC)’ improves the precision from 88.35% to 95.53%, while BiGRU module in ‘XLNet-GRU(RC)’ raises the recall from 94.79% to 95.88%. In contrast, our approach of ‘“XLNet-GRU-TF(RC)’ combines the advantages of ‘TF’ and ‘GRU’, enhancing the classification performance reasonably with a high F1 of 96.34%. By incorporating a residual connection in the model, the “XLNet-GRU-TF” achieves enhanced generalization, resulting in an improved F1 score of 96.34%, compared to the previous score of 94.85%.

Supplementary Figure S1 shows the F1 scores on the validation set and average training loss in relation to each epoch (averaged over the five-fold cross-validation) for URNet-XL and baseline models on the WRD dataset, respectively. Overall, as the iteration epoch increases, all the F1 scores converge. Our URNet-XL model shows the fastest convergence, in which its F1 scores are steady above 95% after the 4th epoch (Supplementary Fig. S1(a)). The high F1 of 96.09% on validation set again indicates the excellent generalization capability of our model in UR identification. In addition, the average training loss represents the fitting ability on the training set. The model’s classification performance improves as the training loss decreases, with the average loss of URNet-XL dropping to < 0.02 after 5 epochs (Supplementary Fig. S1(b)).

We compared the performance of two different ways of processing text data: one is simply concatenation (SC), i.e. linking the three paragraphs in the same ward round record by using the recognizable separator of “< sep>” for XLNet, as one input data for the pre-trained model; the other is batch fusion (BF, our method), i.e. feeding each of the three paragraphs into the model per batch and then fusing the three output results. Note that the input text can be easily overlength in the first method due to the limited GPU resources. Therefore, instead of inputting extra-long combined texts into GPU, we proposed an approach of long text fusion based the second method for computational resource saving.

Furthermore, we explored the effect of maximum sequence lengths on two proposed methods of data processing (SC and BF) as shown in Supplementary Table S3. The method of text encoder fusion completely surpasses the long text concatenating in our cases. In addition, as the useful information is more likely to locate in the front of the text, the method of text encoder fusion outperforms texts with a length of 512 characters compared to those with a length of 1024 characters or longer. The possible reason is that excessively long texts (e.g. 1500 characters) could introduce more noise, which leads to a decrease in the classification performance. In our dataset, the average length of the three paragraphs and that of the concatenated texts are 282 and 777, respectively. The standard deviations for BF remain low, particularly at the 512-character setting, indicating stable performance. In contrast, SC exhibits higher variability with longer sequences, suggesting that excessive text length leads to more inconsistent results. In conclusion, BF with a sequence length of 512 characters offers the best balance of performance and stability.

### Unplanned reoperation cause classification

#### Experimental setups

We took the NEZHA model with 12 layers and 768 of hidden size as our pre-trained language model. We used stratified sampling on the dataset to create the training set, validation set and testing set in a ratio of 75:15:15 (shown in Supplementary Table S4). The initial learning rate for BertAdam was set to be 2e-5, the warmup schedule, the maximum sequence length of token and the batch size were all set as the same as those in 3.1.1. Moreover, 15 epochs per fold were set in five-fold cross-validation training. We also adopted an “early stop” strategy in this task. FGM was employed to enhance the robustness of the model. Finally, we applied stratified sampling to create the training set and the test set, ensuring the consistency of the distribution. We evaluated the performance of our model on the test set by weighted Precision (P), weighted Recall (R), weighted F1 score (F1) and accuracy (ACC). Their formulas are as follows:

27 $$\:{P}_{weighted}=\frac{{\sum\:}_{i=1}^{n}\left({P}_{i}\times\:{w}_{i}\right)}{n}$$

28 $$\:{R}_{weighted}=\frac{{\sum\:}_{i=1}^{n}\left({R}_{i}\times\:{w}_{i}\right)}{n}$$

29 $$\:F{1}_{weighted}=2\cdot\:\frac{{P}_{weighted}\cdot\:{R}_{weighted}}{{P}_{weighted}+{R}_{weighted}}$$

30 $$\:Accuracy=\frac{TP+TN}{TP+FP+TN+FN}$$

where $\:n$ is the number of the total samples, $\:{w}_{i}$ is the proportion of the $\:i$-th category in the total samples, $\:{P}_{i}$ denotes the precision of the $\:i$-th category and $\:{R}_{i}$ represents the recall of the $\:i$-th category.

#### Results

To demonstrate the excellent performance of our method, we compared URNet-GT with a baseline model consisting of BERT with a classifier by the cross-entropy loss evaluation during training, as shown in Table 3. And all methods employed five-fold cross-validation to compute the average performance metrics, thereby minimizing bias and variance, and ensuring more reliable and robust evaluation results on small sample dataset. The URNet-GT model outperforms the baseline model in all respects of performance. Especially, for our small sample dataset of QC, the FGM improves all the results.

**Table 3:** Comparison results of the models.

Method	–				FGM			
	*P*(%)	*R*(%)	F1(%)	ACC(%)	*P*(%)	*R*(%)	F1(%)	ACC(%)
BERT	82.20	83.02	81.58	83.02	84.53	86.49	84.49	86.49
BioBERT	85.62	88.01	84.80	88.01	87.76	88.01	86.87	88.01
ClinicalBERT	86.15	87.12	85.62	87.12	88.30	88.15	87.22	88.15
URNet-GT	89.06 (+ 6.86)	88.68 (+ 5.66)	87.91 (+ 6.33)	88.68 (+ 5.66)	90.17 (+ 5.64)	90.57 (+ 4.08)	89.59 (+ 5.10)	90.57 (+ 4.08)
BERT^(+)^	82.79	85.14	83.71	85.14	86.78	86.79	86.22	86.79
BioBERT^(+)^	86.73	87.45	87.08	87.45	90.02	90.41	90.20	90.41
ClinicalBERT^(+)^	87.58	88.25	87.90	88.25	90.64	91.02	90.82	91.02
URNet-GT^(+)^	92.51 (+ 9.72)	91.89 (+ 6.75)	91.85 (+ 8.14)	91.89 (+ 6.75)	**93.69** **(+ 6.91)**	**93.48** **(+ 6.69)**	**93.37** **(+ 7.15)**	**93.48** **(+ 6.69)**

“FGM” represents the fast gradient method. The values in parentheses represent the improvement in performance. (+) for training on the augmented dataset. The results are based on the average of five-fold cross-validation. Bold numbers highlight the best results in each category

With the same hyperparameters and pre-trained model, we compared the results of different modeling strategies on respective data augmentation methods with raw data, shown in Supplementary Table S5. We can conclude that the focal loss outperforms the cross-entropy loss when dealing with unbalanced samples. Based on the method with BiGRU and multi-head attention mechanism, FGM improves the F1 score from 91.85% to 93.37% and the accuracy from 91.89% to 93.48% respectively in our small sample dataset, although it slightly reduces the results of attention. It demonstrates that the FGM helps to prevent model overfitting in few-shot learning by introducing perturbations. The FGM increases model robustness and generalization, thereby mitigating issues arising from scarce data. Furthermore, in comparison with the results without data augmentation, our method of concatenating the diagnosis with keywords and departments reasonably enhances the classification performance.

Supplementary Figure S2 demonstrates the accuracy on validation set and average training loss in relation to each epoch (averaged over the five-fold cross-validation) for URNet-GT and the other models on the QC dataset. As the number of iterations (epochs) increases, the overall accuracy of each model gradually stabilizes and converges. The accuracy of the URNet-GT model is steady above 90% after the 7th epoch. Although the BERT-base model shows higher accuracy in the early stage, the URNet-GT-fgm finally outperforms it with a faster ascent rate at the later epochs.

The average training loss curve in Supplementary Fig. S2(b) and Supplementary Fig. S2(d) provides a measure of the models’ fitting ability on the training set. As each model tends to coverage after 11th epoch, the URNet-GT-base first reaches a small average loss value of less than 0.25 after 9th epoch. From the convergence point of view, the URNet-GT-base is the best trained one on our task. However, its accuracy in validation set is less than that of the URNet-GT-fgm, since FGM enhances the robustness of the model by adding perturbations during training. BioBERT and ClinicalBERT exhibit strong performance in tasks involving medical terminology and clinical context. However, when processing complex Chinese medical texts, URNet-GT with NEZHA achieves significantly higher F1 scores and accuracy. The superior performance is attributed to NEZHA’s specialized pre-training for Chinese text data. NEZHA’s advanced capabilities in managing complex, multi-class classification within Chinese medical texts enable URNet-GT to surpass these models on both standard and augmented datasets.

The URNet-GT model demonstrates a clear advantage in both F1 score and accuracy across all evaluation folds (shown in Supplementary Table S6 and Supplementary Table S7). The URNet-GT-fgm^(+)^ version achieves the highest performance, with an impressive average F1 score of 93.37% ± 0.19% and accuracy of 93.48% ± 0.16%, significantly outperforming all other models. This result highlights the effectiveness of combining FGM training with an augmented dataset, which enables the model to capture key features more accurately and reduces the risk of errors in classifying long medical texts. Even the URNet-GT-fgm model, without the augmented data, still shows strong results, with an average F1 score of 89.59% ± 0.20% and accuracy of 90.57% ± 0.15%, demonstrating the substantial benefits of FGM training in performance improvement. URNet-GT’s consistent performance underscores its robustness and superior ability to handle complex medical language. The combination of FGM and augmented data in the URNet-GT-fgm^(+)^ version proves to be particularly effective, making it the top choice for accurately classifying UR medical records and achieving higher overall performance in both F1 score and accuracy.

To thoroughly investigate the performance of URNet-GT, we generated a heatmap illustrating the multi-class classification results of the URNet-GT model on the testing set (shown in Supplementary Fig. S3). The sub-diagonal of the matrix represents correctly classified instances by the model. The off-diagonal areas indicate the number of misclassifications, reflecting the degree of confusion between different classes. The model outperforms on most categories, such as “thrombus”, “subsidiary injury of an operation”, “cerebral edema cerebral embolism”, “flap crisis” and “pathology”, with no incorrect assignments. These categories have distinct text features, making them highly distinguishable. However, “bleeding” and “infection” are misclassified. For example, an instance of “flap crisis” is mistakenly classified as “bleeding”. This indicates a degree of similarity in the text descriptions between the some of the categories, making the model confusing.

### Clinical validation

To validate the core assumptions of UR-Net, we recruited clinical experts of Peking University Shenzhen Hospital for systematic evaluation: Three attending surgeons with over 10 years of clinical experience were invited. A total of 200 WRDs (50 UR cases, 150 non-UR cases) and 100 QC texts (covering 12 reasons) were randomly sampled from the dataset for blinded validation. The validation focused on three dimensions:

- Sufficiency of WRD for UR Identification: Experts rated whether WRDs contained sufficient information to determine UR using a 5-point Likert scale^72^ (where 5 = “fully sufficient”).
- Consistency of Attention Mechanisms with Clinical Reasoning: We compared the top-10 tokens with the highest attention weights (extracted from the model’s attention layer) against “clinically critical sentences” annotated by experts to calculate token overlap rates.
- Accuracy of UR Cause Extraction: Experts independently judged whether the model’s UR reason predictions matched clinical diagnostic criteria.

As a result, the average rating for WRD information sufficiency was ($\:4.72\pm\:0.35$). Among all samples, 92% received a score $\:\ge\:$ 4, confirming that WRDs contain adequate information for UR prediction and supporting the model’s core assumption. The token overlap rate between the model’s attention-weighted top tokens and experts’ clinically critical sentences reached $\:91.6\pm\:4.2\%$. For instance, in cases of “surgical site infection”, the model consistently emphasized tokens like “purulent discharge”, “fever”, and “incision redness”—terms that aligned with experts’ focus on infectious manifestations. Finally, the UR cause extraction agreement rate between expert judgments and model predictions was $\:91.3\pm\:3.8\%$, significantly higher than the random baseline by 8.3%.

High scores for WRD sufficiency verify that batch fusion method effectively preserves clinically relevant information. The strong overlap in attention tokens demonstrates that the UR-Net’s attention mechanisms capture semantically critical terms consistent with clinical reasoning—addressing concerns about the interpretability and clinical grounding of deep learning features. Discrepancies (observed in 3 complex cases with combined etiologies like “bleeding + infection”) were attributed to the model’s current limitation in multi-label classification, which will be prioritized in future work (e.g., integrating multi-label loss.

## Discussion

In addition to the identification and cause analysis, we also analyzed the temporal distribution of surgeries that tend to lead to UR using structured numerical data. Supplementary Figure S4(a) shows that most surgeries are typically performed at 9:00 am, with the number of surgeries gradually decreasing. Meanwhile, the surgeries at 9:00 am have the highest incidence of URs (shown in Supplementary Fig. S4(b)). One possible reason for this correlation is that the more surgeries at the beginning of working time induces the more work pressure on doctors, which may lead to lower quality operation. Furthermore, we counted the number of occurrences of URs by the month from 2015 to 2021. No obvious pattern of UR monthly occurrence was observed in Supplementary Fig. S5.

Counting the number of URs by department, we found that orthopedics and spinal surgery, gastrointestinal surgery and department of stomatology had the highest number of URs (shown in Supplementary Table S8). Additionally, the top three UR causes are bleeding, leakage or fistula or obstruction, and infection, corresponding to 352, 103 and 87, respectively (shown in Table 1). These statistical results are similar to those conclusions published recently. For example, Li et al.^15^ observed similar trends in a third-class hospital setting. Wang et al.^65^ found comparable causes and proposed countermeasures for UR in a tertiary general hospital. Ouyang et al.^66^ conducted a large-scale study with over 35,000 patients, identifying common causes and risk factors for URs after spine surgery. Our analysis suggests that certain departments have a high probability of URs due to the high difficulty and large number of their surgeries. Besides our result, bleeding, postoperative fistula and infection have also been considered as the main causes due to incomplete hemostasis, inadequate postoperative supervision and care, or poor wound healing ability of patients^67^. In addition to text records, the patient’s personal information has been used as a considerable factor for the UR determination. For example, the probability of UR is related to the patient’s age, which gives the maximum over 60 or 65 years^6^. This finding aligns with the study by Zou and Chen^68^, who also noted that elderly patients are more prone to complications after surgery due to physiological aging. Therefore, age should be considered when optimizing the model in the future.

The irrelevant content in medical target text induces noise that significantly impacts model performance. To mitigate this issue, noise reduction can be achieved through data preprocessing techniques to filter out such texts. In our work, the integration of attention mechanisms with long medical text processing can efficiently extract crucial information and yield positive outcomes in our case, e.g. time-saving compared to manual processing. Moreover, in consideration of its reliability, our approach is heuristic for long medical text processing with the potential for generalization to other domains. Our hybrid approach (neural architecture + handcrafted feature guidance) aligns with emerging medical NLP paradigms that address data noise through feature fusion. For instance, Taşcı^69^ combines handcrafted acoustic features with deep learning significantly improved depression recognition from speech, and the work validates the power feature fusion in clinical AI.

The standardization of record-keeping significantly impacts the classification performance of models. Omissions or errors in records can prevent the model from accurately capturing disease characteristics and associations, leading to deviations from actual classification outcomes. If scribes arbitrarily use medical terms or confuse concepts during record writing, the model may misinterpret the information, resulting in biased classification results. Although NLP technology can mitigate noise and interference through preprocessing, hospitals must establish strict record-keeping standards. This ensures that medical texts are accurate and standardized, providing cleaner and more reliable training data for models.

To assess the scalability of the UR-Net framework, we evaluated its training time and hardware requirements using hospital-grade equipment (e.g., NVIDIA Tesla A100 GPU 80GB). The URNet-XL model training took about 1.25 h on 15 epochs, and the URNet-GT model training took about 1 h on 15 epochs (with 5 folds per epoch), with the largest computational cost stemming from the text length. Inference time for each document was found to be 1 s, ensuring timely processing in clinical environments. As for scalability, the model performed well on larger datasets, but optimizations such as parallel processing may be needed for even larger clinical datasets.

While our dataset from Peking University Shenzhen Hospital demonstrates UR-Net’s efficacy, we acknowledge the inherent limitations in generalizing findings across diverse clinical settings. To address this, we supplemented our analysis with critical assessments of temporal stability and documentation adaptability. We validated UR-Net across non-overlapping time cohorts: 2015–2019 and 2020–2021. Performance remained stable with minimal decay: UR identification of URNet-XL F1-score decreased by only 0.63% (96.52% to 95.91%), and cause classification of URNet-GT weighted F1-score dropped by 0.72% (93.54% to 92.87%). This confirms the model is not overfitted to time-specific patterns.

Since the hospital’s privacy policy, we are unable to obtain additional data resources. This limitation posed challenges in data acquisition and model validation. Although the current study focuses on data from one hospital, we plan to validate UR-Net on datasets from multiple institutions upon ethical approval to enhance its broader generalizability. Nevertheless, we have made every effort to maximize the accuracy and stability of our model with the available data. By employing advanced deep learning techniques and NLP methods, we aim to ensure the models’ generalization capability and practical applicability. Furthermore, we maintain a high level of respect for data privacy and security, with all data processing and analysis strictly adhering to relevant regulations and ethical guidelines.

To mitigate risks, we recommend incorporating clinician oversight to validate model predictions before implementation. Specifically, the model’s predictions should undergo clinician validation before implementation to mitigate risks from false positives (e.g., unnecessary interventions) or false negatives (e.g., delayed responses to complications). When integrated into EHR systems, UR-Net can thus automatically and safely analyze surgical records and clinical notes to identify high-risk cases, enabling clinician-supervised early interventions. For example, in post-operative monitoring, UR-Net’s real-time analysis of clinical notes combined with mandatory physician sign-off could reduce the risk of preventable URs and enhance patient safety.

Supplementary Figure S6 demonstrates a clinical workflow of UR-Net for real-time surgical complication diagnosis. The attention mechanism demonstrates clinical alignment, where the maximum weight assigned to the “infection” token corresponds directly with surgical diagnostic reasoning. Additionally, UR-Net has the potential to streamline the workflow of quality assurance committees by generating detailed, data-driven reports on surgical outcomes. These reports can help identify recurring pattern or systemic issues in surgical procedures, facilitating targeted improvements in hospital practices. Furthermore, validated predictions allow administrators to forecast UR trends and allocate resources efficiently while maintaining the accountability framework essential for clinical deployment.

To further operationalize the framework, UR-Net syncs with EHRs via a secure API to auto-ingest daily de-identified WRDs and QC texts. URNet-XL performs real-time inference to generate a UR risk score (0–100), driving a tiered workflow: scores < 30 are auto-tagged “low-risk” (no manual intervention); 30–70 trigger quality control expert review; and scores > 70 alert attending physicians with attention-weighted critical tokens (e.g., “postoperative bleeding”) to prioritize review. If UR-Net incorrectly flags a low-risk patient as high-risk, it leads to unnecessary interventions, reviews, and resource use (though no direct patient harm). Conversely, missing a truly high-risk patient (false negative) delays critical treatment, risking adverse outcomes like infection or reoperation. To address this, a clinician-in-the-loop system enables UR-Net to correct predictions, with de-identified correction data used for monthly minibatch fine-tuning to avoid catastrophic forgetting and reduce false positives/negatives over time.

## Conclusion and extensions

In conclusion, we propose UR-Net, a framework for the identification and cause extraction of URs, which comprises two components: URNet-XL and URNet-GT. The innovative architecture of URNet-XL addresses the issue of context information loss in traditional models when processing long texts by dividing the text into three segments and combining them post-encoding. This multi-segment encoding approach, coupled with the utilization of BiGRU and Transformer layers, enhances the model’s capability to capture long-range contextual dependencies. URNet-XL offers domain-specific adaptations to handle complex medical texts, incorporating XLNet models tailored for long texts and advanced context modeling through BiGRU integration to mitigate the challenge of limited medical data. They significantly boost the model’s semantic understanding of long texts while preserving high computational efficiency. URNet-GT addresses the small sample classification challenge of extracting the UR causes from Chinese quality control texts. The model builds on the NEZHA pre-trained model, which is particularly effective for processing Chinese medical text, and the texts are further processed using a BiGRU and a transformer encoder for comprehensive feature extraction. The URNet-GT enhances it with the FGM to improve the robustness. The combination ensures the model captures both local and global context within the texts, enhancing the classification accuracy.

While BERT and its variants (e.g., BioBERT and ClinicalBERT) have demonstrated strong performance in many NLP tasks, UR-Net integrates novel components that make it more suitable for identifying URs and analyzing their causes in medical texts. BioBERT and ClinicalBERT are well-suited for biomedical and clinical tasks, respectively. However, despite the advantages of BioBERT and ClinicalBERT in handling specialized medical languages, UR-Net demonstrates clear superiority in this study. By utilizing UR-Net, medical quality control departments can monitor and evaluate URs in a faster and more effective way, allowing for hospitals prompt and accurate responses to URs, potentially reducing their occurrence of URs in the medical process.

Although our study primarily focuses on data augmentation to enhance model performance, recent advances in meta-learning and domain adaptation have shown great promise in addressing challenges associated with limited data availability and improving model generalization. In the future research, these methods could be systematically investigated to further improve performance, particularly in scenarios where labeled data is scarce or unavailable. Additionally, techniques such as domain adaptation could be explored to facilitate model transferability and refinement when applied to novel tasks or datasets. Incorporating these approaches would provide a more robust solution for UR cause classification and expand the applicability of the proposed framework to broader and more diverse contexts.

In the future, we will extend our framework to combine batch fusion with keyword extraction to reduce medical text length without losing information, and novel feature selection and elimination methods to reduce manual intervention and potential biases in the analysis process. Incorporating a richer set of structured data features (patient age, gender, and surgery name, etc.) and the surgery history of patients into the UR identification model can enhance its performance in identifying patients at higher risk. Thus, the framework can adapt to evolving data patterns and reduce false positives. This improvement will facilitate a deeper understanding of the underlying causes of URs and contribute to offering enhanced support and guidance for medical quality control. Furthermore, by establishing collaborations with more hospitals to access a larger volume of surgery data quality control data, the generalizability of the model can be substantially enhanced. This expansion in data diversity and volume will enable the model to perform with increased reliability across a broad spectrum of healthcare settings, thereby broadening its applicability and impact in the medical domain.

## Supplementary Information

Below is the link to the electronic supplementary material.

Supplementary Material 1

## Data Availability

The datasets analyzed during the current study are not publicly available due to privacy but are available from the corresponding author on reasonable request.
