# Supplementary material for: Identification and cause analysis on unplanned reoperations by text classification approach
Source: Sci Rep. 2025 Nov 10;15:39246. doi: 10.1038/s41598-025-22791-9 (PMC12603117; doi:10.1038/s41598-025-22791-9)
Supplement: Supplementary file 1 — Supplementary Material 1 [file 41598_2025_22791_MOESM1_ESM.docx]

**Identification and Cause Analysis on Unplanned Reoperations by Text Classification Approach**

Zhancheng Liang^1,‡^, Wenyang Huang^1,‡^ , Hongyu Xu^1^, Zhenkun He^1^, ChunQiu Yuan^1^, Yan Liang^2^, Qiuquan Guo^1^, Tianzhao Liu^3,*^, and Peipei Jia^1,*^

^1^ University of Electronic Science and Technology of China, China

^2^ Wuyi University, China

^3^ Peking University Shenzhen Hospital, China

* liutianzhao@vip.qq.com

* jiapeipei@uestc.edu.cn

**SUPPLEMENTARY INFORMATION**


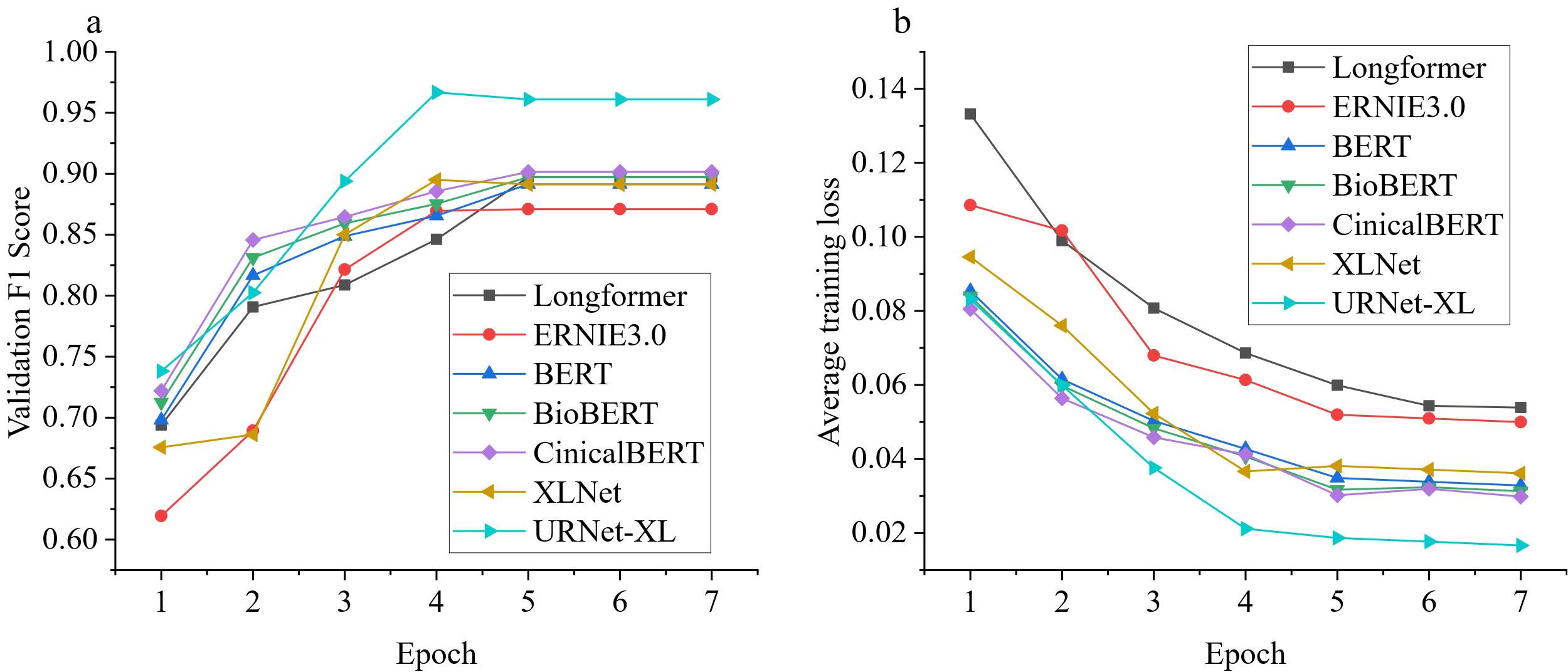


**Supplementary Figure S1.** Model convergence analysis on Ward Round Document (WRD) data: Validation F1 score and average training loss across epochs.​​ (a) ​Validation F1 score progression​ (range: 0.60–1.00) demonstrates classification performance improvements as training advances. URNet-XL consistently outperforms baselines (XLNet, BioBERT, ClinicalBERT, BERT, ERNIE3.0, Longformer), achieving >0.95 F1 by epoch 5. (b) ​Average training loss dynamics​ (range: 0.02–0.14) reveal optimization efficiency. URNet-XL converges rapidly (<0.04 loss by epoch 2), while BioBERT/ERNIE3.0 exhibit higher volatility (note label overlap in ClinicalBERT/BERT entries). Collectively, these trends validate URNet-XL’s accelerated convergence, stable generalization, and lower susceptibility to overfitting compared to benchmark models.


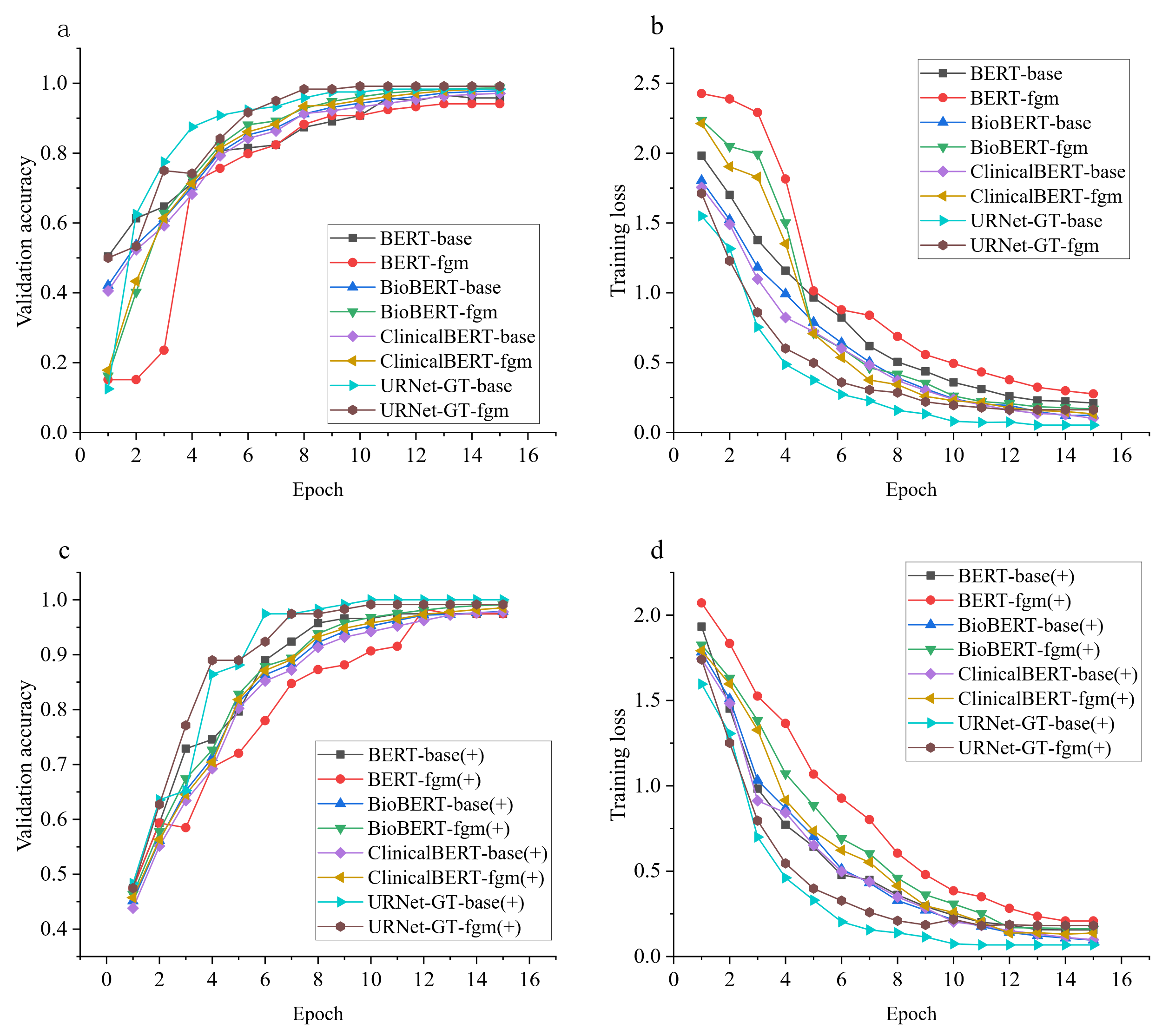


**Supplementary Figure S2.** Impact of Fast Gradient Method (FGM) and data augmentation on UR cause classification.​​ (a–b) ​Baseline dataset: (a) Validation accuracy (range: 0.65–0.98) and (b) average training loss (range: 0.05–0.30) across epochs for models: ​BERT-base, ​BioBERT-base, ​BioBERT-large, and ​URNet-GT. (c–d) ​Augmented dataset (+)​: (c) Validation accuracy and (d) training loss under identical settings. URNet-GT consistently achieves the highest accuracy (>95%) and fastest convergence. FGM reduces loss volatility across all models, while augmentation (+) elevates peak accuracy by 3–7%.


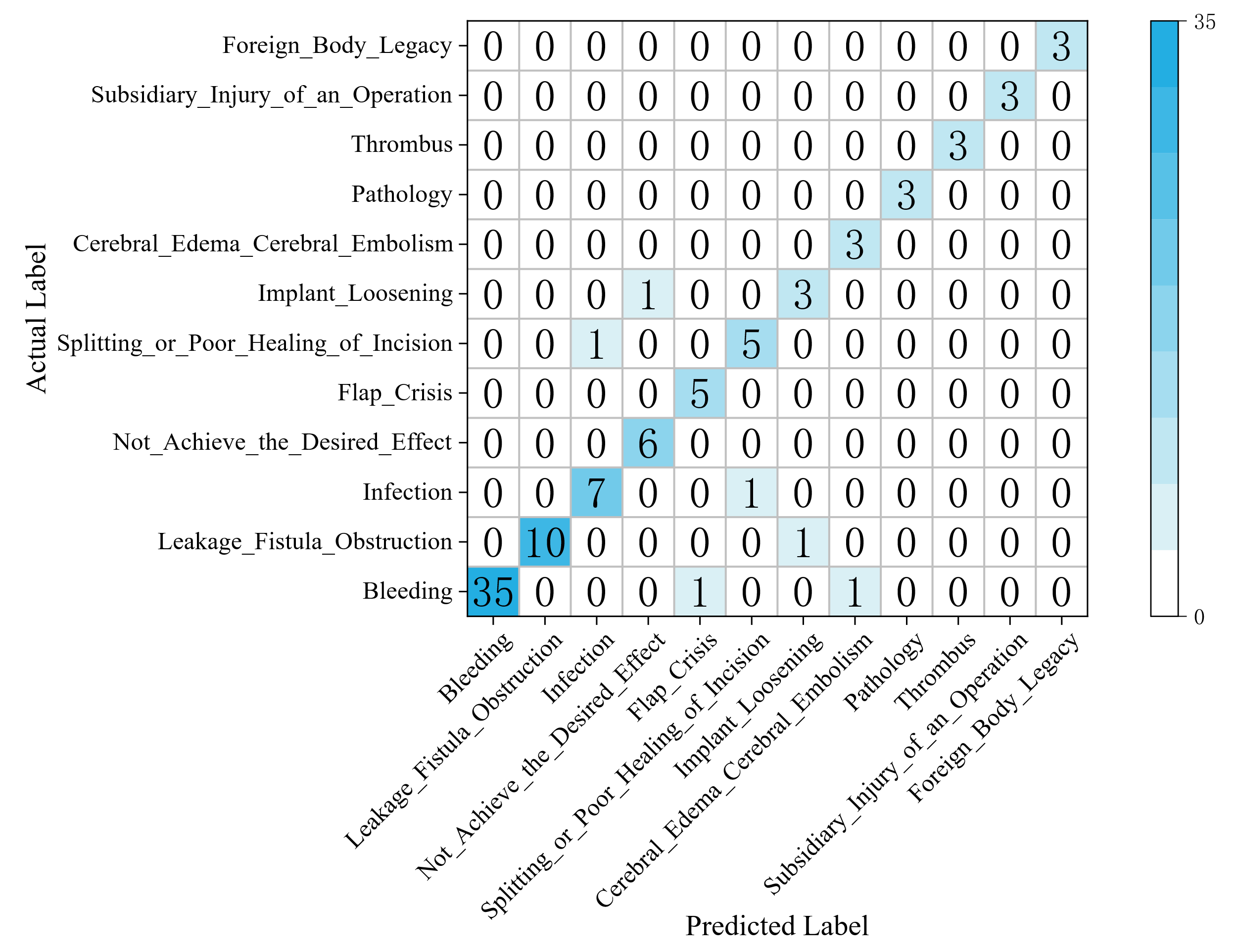


**Supplementary Figure S3.** Confusion Matrix Heatmap for the URNet-GT model performance on the testing set. Cell values denote sample counts with gradient coloring (white $\to$ navy blue) reflecting prediction frequency.

- High Precision in Critical Events: Bleeding (row 1: 35/37 correct; precision=94.6%) exhibits minimal false negatives (<3%), though self-misclassification suggests potential overfitting.
- High-Risk Misclassifications: Infection (row 2) shows systematic confusion with Not Achieve the Desired Effect (7 cases), indicating clinically critical error patterns.
- ​Low-Frequency Limitations: Rare complications (<5 samples, e.g., Foreign Body Legacy, Subsidiary Injury) demonstrate perfect recall but suffer from sparse representation.
- ​Diagnostic Visual Cues: Darker diagonals validate model reliability for Cerebral Edema and Thrombus; off-diagonal blues reveal confusion between Implant Loosening and Pathology.


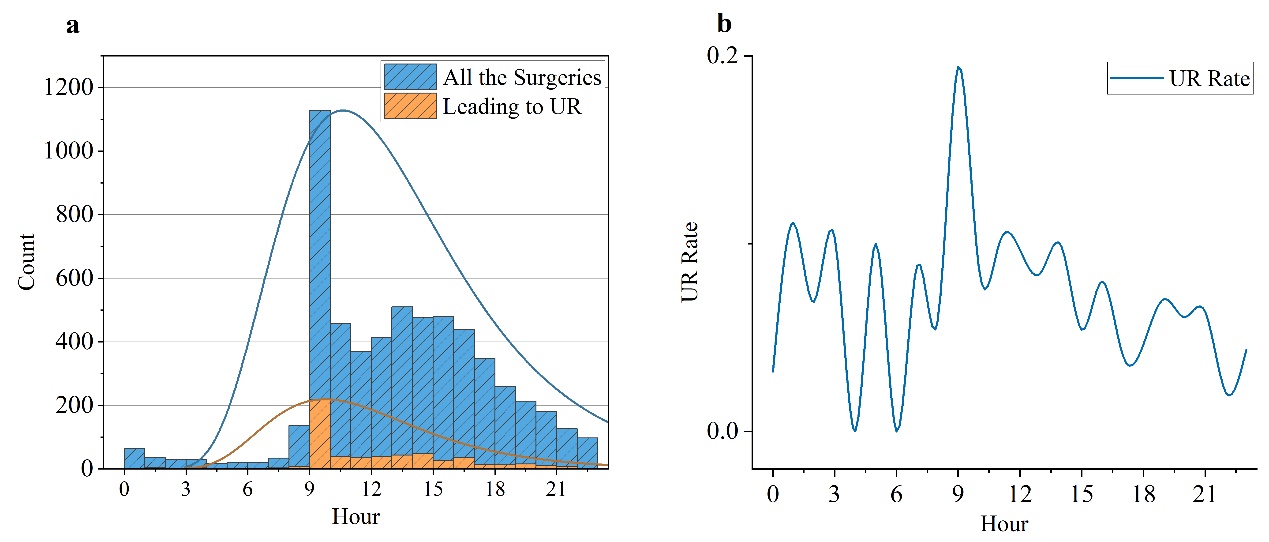


**Supplementary Figure S4.** The relationship between time and unplanned reoperations (URs). (a) ​Surgery volume and UR risk association: Blue bars (>3,000 total surgeries; peak: 1,200 cases at 9:00) represent hourly operation counts. Overlaid orange line tracks UR proportion among procedures (range: 0.0$-$0.08). Note UR-risk escalates during peak volumes (09:00$-$15:00) but stabilizes during nocturnal periods (00:00$-$06:00) despite low surgical activity. (b) ​Hourly UR incidence dynamics: Blue line plots UR rate per procedure (range: 0.0$-$0.15), revealing: (1) bimodal peaks at 09:00 and 17:00, and (2) near-zero nocturnal rates (03:00$-$04:00). Incidence consistently remains <0.10 during afternoon (15:00$-$19:00). Strong positive correlation (r≈0.82) between surgery volume and UR risk suggests resource strain elevates complications, while nighttime stability implies adequate staffing mitigates UR emergence.


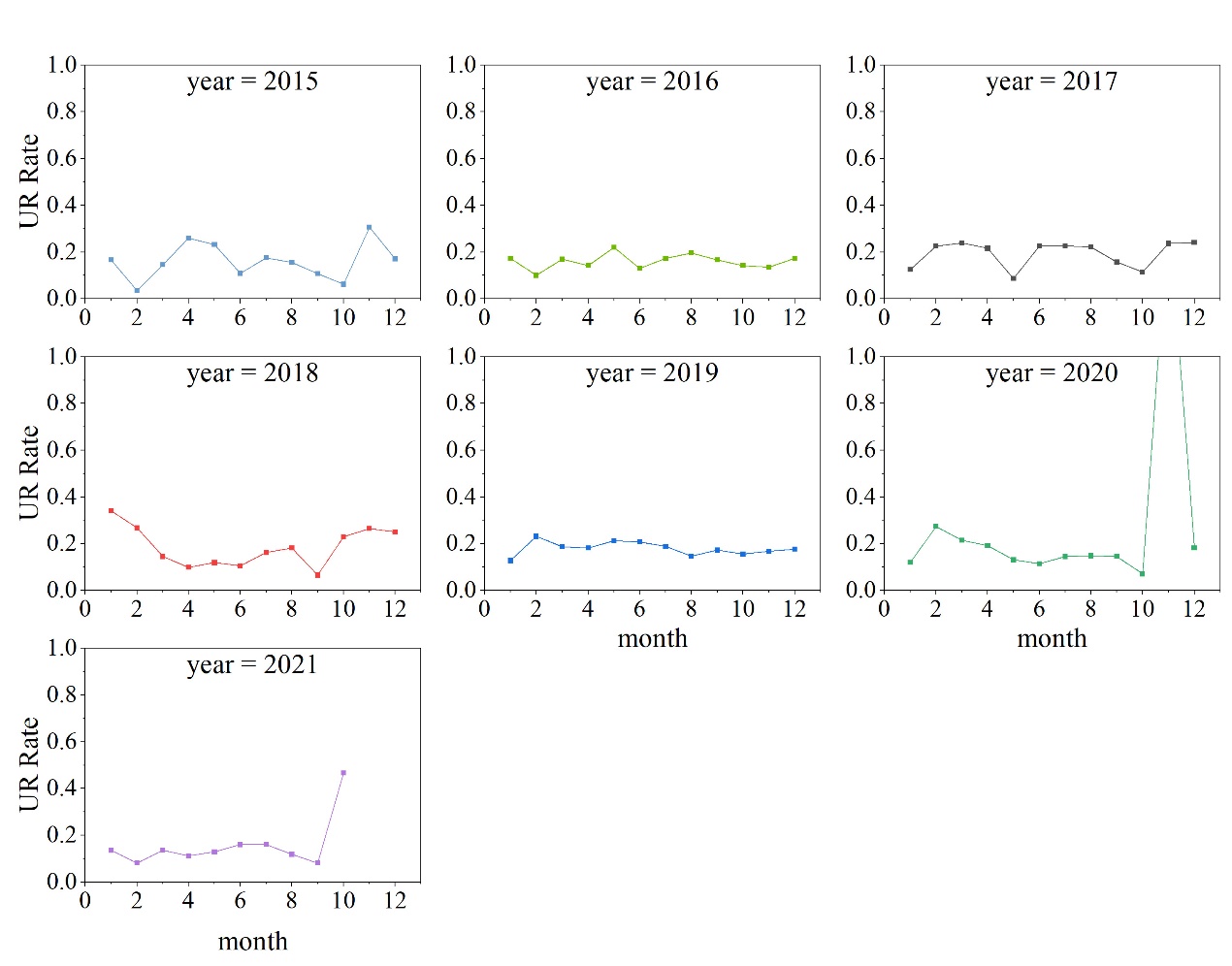
 **Supplementary Figure S5.** Annual trends in the occurrence of URs from 2015 to 2021. Cyclical peaks in spring (March$-$April) and autumn (September$-$November). The 7-year visualization reveals seasonality in surgical safety hazards.


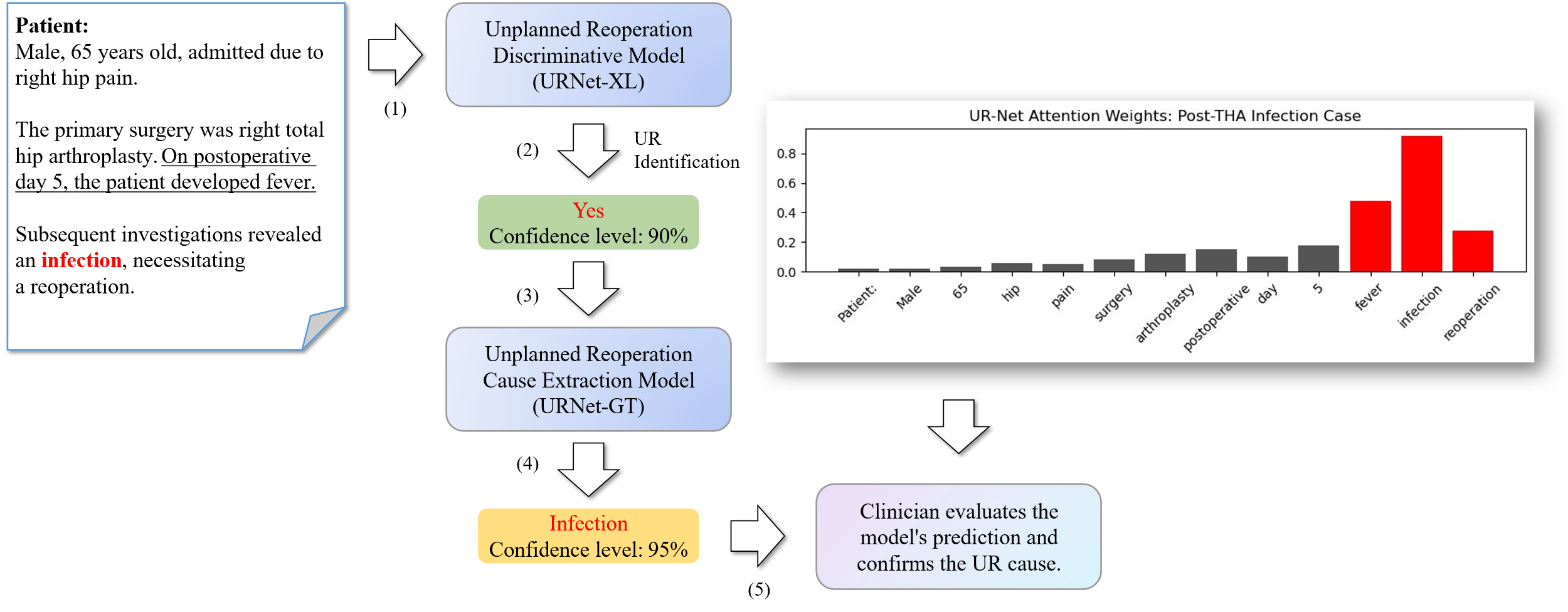


**Supplementary Figure S6.** Clinical workflow demonstration of UR-Net for real-time surgical complication diagnosis with an attention weights heatmap.​​ Case example: 65-year-old male status post right total hip arthroplasty, reoperated on postoperative day 5 for infection. Input: Ward round document (WRD) details clinical deterioration (fever/pain); URNet-XL: Detects unplanned reoperation (UR) with ​90% confidence​ $\to$ Triggers automated transfer to cause analysis; URNet-GT: Processes quality control (QC) text, identifies “Infection” as primary cause (95% confidence); Clinical integration: Surgeon validates prediction against intraoperative findings (e.g., purulent drainage, culture results).

| Dataset | Total Samples | Positive Samples | Negative Samples |
| --- | --- | --- | --- |
| Training | 1860 | 493 | 1367 |
| Validation | 327 | 89 | 238 |
| Testing | 327 | 96 | 231 |

**Supplementary Table S1.** Distribution of ward round document (WRD) samples across training, validation, and testing splits for UR identification.​​ Dataset stratification: Total (n=2514) → Training (n=1860, 74.0%), Validation (n=327, 13.0%), Testing (n=327, 13.0%). Positive samples: Documented instances of unplanned reoperations (URs) → Prevalence: Training: 26.5% (493/1860), Validation: 27.2% (89/327), Testing: 29.4% (96/327). Negative samples: Records of planned/elective reoperations → Ratio to positives: Training (2.8:1), Validation (2.7:1), Testing (2.4:1).

Note: Distribution preserves UR incidence consistency ($\pm3\%$) across splits, mitigating representation bias.

| PTM | Method | P (%) | R (%) | F1 (%) | AUC (%) |
| --- | --- | --- | --- | --- | --- |
| XLNet | – | 88.35 | 94.79 | 91.46 | 94.80 |
|  | TF | 93.28 (+4.93) | 95.76 (+0.97) | 94.50 (+3.04) | 96.60 (+1.80) |
|  | GRU | 93.81 (+5.46) | 94.79 (+0.00) | 94.30 (+2.84) | 96.10 (+1.30) |
|  | GRU+TF | 93.88 (+5.53) | 95.83 (+1.04) | 94.85 (+3.39) | 96.62 (+1.82) |
|  | TF (RC) | 95.53 (+7.18) | 94.68 (-0.11) | 95.10 (+3.64) | 97.02 (+2.22) |
|  | GRU (RC) | 93.00 (+4.65) | **95.88 (+1.09)** | 94.42 (+2.96) | 96.56 (+1.76) |
|  | GRU+TF (RC) | **96.84 (+8.49)** | 95.83 (+1.04) | **96.34 (+4.88)** | **97.86 (+3.06)** |

**Supplementary Table S2.** Ablation experiment results of different methods for UR identification. “PTM” for pre-trained model. “–” for classification by CLS embedding only. Bold numbers indicate the best results. Transformer encoder (TF) and BiGRU (GRU) individually boost F1 by +2.84–3.04% versus baseline (91.46% → 94.30–94.50%). Combined GRU+TF delivers +3.39% F1 (94.85%) without residual connections (RC). GRU+TF(RC) achieves ​peak performance: 96.84% precision (+8.49%), 96.34% F1 (+4.88%), and 97.86% AUC (+3.06%) – outperforming all other configurations.

| Method | Maximum Sequence Length | P (%) | R (%) | F1 (%) | AUC (%) |
| --- | --- | --- | --- | --- | --- |
| SC | 777 (average) | 73.65 ± 1.22 | 63.77 ± 1.18 | 68.35 ± 1.10 | 78.76 ± 1.35 |
|  | 2048 (> 96%) | 68.24 ± 1.10 | 70.46 ± 1.08 | 69.33 ± 1.05 | 80.34 ± 1.15 |
| BF | 282 (average) | 77.98 ± 1.15 | 88.54 ± 1.12 | 82.93 ± 1.10 | 89.08 ± 1.22 |
|  | 512 (> 89%) | **96.84** ± 0.84 | **95.83** ± 0.76 | **96.34** ± 0.82 | **97.86** ± 0.71 |
|  | 900 (> 96%) | 94.79 ± 0.88 | 94.79 ± 0.85 | 94.79 ± 0.91 | 96.31 ± 0.67 |
|  | 1024 (> 97%) | 88.66 ± 0.91 | 89.58 ± 0.87 | 89.81 ± 0.84 | 92.41 ± 0.78 |
|  | 1200 (> 98%) | 80.34 ± 1.02 | 97.92 ± 0.72 | 88.26 ± 0.85 | 93.98 ± 0.67 |

**Supplementary Table S3.** Sequence length optimization for URNet-XL architecture: Performance comparison of batch fusion (BF) vs. simple concatenation (SC) across document length thresholds.​ BF for batch fusion method. SC for simple concatenation method. “>” indicates that the maximum sequence length setting covers the number of WRD text length. “Average” refers to the average text length. Bold numbers indicate the best results. The values are presented as average value ± standard deviation (Std) from the five-fold cross-validation. XLNet-GRU-TF(RC) model tested under maximum sequence lengths (columns) mirroring ward round document (WRD) characteristics. At clinically optimal 512-length (covers >89% WRDs), BF achieves ​peak metrics: F1 96.34% (±0.82), AUC 97.86% (±0.71), outperforming SC (F1 69.33%) by 27.01%. BF performance declines beyond 512-length (F1: 94.79% $@$900$\to$88.26% $@$1200), revealing computational saturation. SC struggles at average lengths (777: F1 68.35%$\pm$1.10), proving BF mitigates context fragmentation.

| Cause | Training | Validation | Testing |
| --- | --- | --- | --- |
| Bleeding | 281 | 36 | 35 |
| Leakage, Fistula, Obstruction | 82 | 11 | 10 |
| Infection | 70 | 9 | 8 |
| Not Achieve the Desired Effect | 56 | 7 | 7 |
| Flap Crisis | 34 | 6 | 6 |
| Splitting or Poor Healing of Incision | 33 | 6 | 6 |
| Implant Loosening | 21 | 5 | 4 |
| Cerebral Edema, Cerebral Embolism | 16 | 4 | 4 |
| Pathology | 13 | 2 | 3 |
| Thrombus | 10 | 2 | 3 |
| Subsidiary Injury of an Operation | 10 | 2 | 3 |
| Foreign Body Legacy | 10 | 2 | 3 |

**Supplementary Table S4.** Stratification of the quality control (QC) dataset: Case counts per complication cause category across training, validation, and testing partitions.

| Method | Loss  Fct | - | | | | FGM | | | |
| --- | --- | --- | --- | --- | --- | --- | --- | --- | --- |
|  |  | P  (%) | R  (%) | F1  (%) | ACC  (%) | P  (%) | R  (%) | F1  (%) | ACC  (%) |
| Base | CEL | 83.39 | 86.79 | 84.72 | 86.79 | 84.85 | 86.79 | 85.14 | 86.79 |
| Base+GRU |  | 85.55  (+2.16) | 86.79  (+0.00) | 85.74  (+1.02) | 86.79  (+0.00) | 86.54  (+1.69) | 88.68  (+1.89) | 87.27  (+2.13) | 88.68  (+1.89) |
| Base+TF |  | **88.35**  **(+4.96)** | **87.74**  **(+0.95)** | **86.97**  **(+2.25)** | **87.74**  **(+0.95)** | 88.72  (+3.87) | **89.62**  **(+2.83)** | **88.68**  **(+3.54)** | **89.62**  **(+2.83)** |
| Base+GRU+TF |  | 88.08  (+4.69) | **87.74**  **(+0.95)** | 86.74  (+2.02) | **87.74**  **(+0.95)** | **89.65**  **(+4.80)** | 88.68  (+1.89) | 87.64  (+2.50) | 88.68  (+1.89) |
| Base | FL | 86.60 | 87.74 | 86.53 | 87.74 | 87.34 | 89.62 | 87.85 | 89.62 |
| Base+GRU |  | 87.41  (+0.81) | 88.68  (+0.94) | 87.81  (+1.28) | 88.68  (+0.94) | 88.25  (+0.91) | **90.57**  **(+0.95)** | 89.09  (+1.24) | **90.57**  **(+0.95)** |
| Base+TF |  | 87.94  (+1.34) | **89.62**  **(+1.88)** | **88.31**  **(+1.78)** | **89.62**  **(+1.88)** | 89.36  (+2.02) | 89.62  (+0.00) | 88.47  (+0.62) | 89.62  (+0.00) |
| Base+GRU+TF |  | **89.06**  **(+2.46)** | 88.68  (+0.94) | 87.91  (+1.38) | 88.68  (+0.94) | **90.17**  **(+2.83)** | **90.57**  **(+0.95)** | **89.59**  **(+1.74)** | **90.57**  **(+0.95)** |
| Base^(+)^ | CEL | 84.06 | 87.16 | 85.39 | 87.16 | 87.75 | 89.19 | 88.19 | 89.19 |
| Base^(+)^+GRU |  | 87.12  (+3.06) | 89.19  (+2.03) | 88.03  (+2.64) | 89.19  (+2.03) | 89.20  (+2.59) | 89.86  (+0.67) | 89.18  (+0.99) | 89.86  (+0.67) |
| Base^(+)^+TF |  | 89.65  (+5.59) | 88.51  (+1.35) | 88.15  (+2.76) | 88.51  (+1.35) | 90.34  (+2.59) | 89.86  (+0.67) | 89.79  (+1.60) | 89.86  (+0.67) |
| Base^(+)^+GRU+TF |  | **90.50**  **(+6.44)** | **89.86**  **(+2.70)** | **89.95**  **(+4.56)** | **89.86**  **(+2.70)** | **92.32**  **(+4.57)** | **91.89**  **(+2.70)** | **91.96**  **(+3.77)** | **91.89**  **(+2.70)** |
| Base^(+)^ | FL | 84.48 | 86.49 | 85.26 | 86.49 | 88.18 | 87.84 | 87.84 | 87.84 |
| Base^(+)^+GRU |  | 87.53  (+3.05) | 88.51  (+2.02) | 87.73  (+2.47) | 88.51  (+2.02) | 89.48  (+1.30) | 89.86  (+2.02) | 89.36  (+1.52) | 89.86  (+2.02) |
| Base^(+)^+TF |  | 89.65  (+5.17) | 89.19  (+2.70) | 89.11  (+3.85) | 89.19  (+2.70) | 90.85  (+2.67) | 90.54  (+2.70) | 90.50  (+2.66) | 90.54  (+2.70) |
| Base^(+)^+GRU+TF |  | **92.51**  **(+8.03)** | **91.89**  **(+5.40)** | **91.85**  **(+6.59)** | **91.89**  **(+5.40)** | **93.69**  **(+5.51)** | **93.48**  **(+5.64)** | **93.37**  **(+5.53)** | **93.48**  **(+5.64)** |

**Supplementary Table S5.** Ablation experiment results of different models and datasets. Base for fine-tuning on the original dataset only by NEZHA and a linear layer classifier; Base^(+)^ for fine-tuning on the augmented dataset only by NEZHA and a linear layer classifier; “Loss Fct” for loss function; CEL for cross-entropy loss; FL for focal loss; TF for transformer encoder module; GRU for Bi-GRU. Bold numbers indicate the best results.

| Method | Fold 1 (%) | Fold 2 (%) | Fold 3 (%) | Fold 4 (%) | Fold 5 (%) | Mean ± Std (%) |
| --- | --- | --- | --- | --- | --- | --- |
| BERT | 81.35 | 81.75 | 81.58 | 81.89 | 81.35 | 81.58 ± 0.24 |
| BioBERT | 84.58 | 84.88 | 84.72 | 85.05 | 84.77 | 84.80 ± 0.18 |
| ClinicalBERT | 85.45 | 85.75 | 85.62 | 85.80 | 85.50 | 85.62 ± 0.15 |
| URNet-GT | 87.63 | 87.89 | 87.91 | 88.16 | 87.94 | 87.91 ± 0.19 |
| BERT^(+)^ | 83.56 | 83.50 | 83.71 | 83.92 | 83.85 | 83.71 ± 0.18 |
| BioBERT^(+)^ | 86.85 | 87.15 | 87.08 | 87.25 | 87.05 | 87.08 ± 0.15 |
| ClinicalBERT^(+)^ | 87.60 | 88.00 | 87.90 | 88.20 | 87.80 | 87.90 ± 0.22 |
| URNet-GT^(+)^ | 91.60 | 92.00 | 91.85 | 92.04 | 91.77 | 91.85 ± 0.18 |
| BERT-fgm | 84.24 | 84.53 | 84.49 | 84.86 | 84.34 | 84.49 ± 0.24 |
| BioBERT-fgm | 86.92 | 86.88 | 86.97 | 87.35 | 86.73 | 86.97 ± 0.23 |
| ClinicalBERT-fgm | 87.06 | 86.96 | 87.19 | 87.46 | 87.42 | 87.22 ± 0.22 |
| URNet-GT-fgm | 89.37 | 89.65 | 89.59 | 89.88 | 89.45 | 89.59 ± 0.20 |
| BERT-fgm^(+)^ | 85.88 | 86.21 | 86.34 | 86.50 | 86.17 | 86.22 ± 0.23 |
| BioBERT-fgm^(+)^ | 89.90 | 90.35 | 90.29 | 90.42 | 90.06 | 90.20 ± 0.22 |
| ClinicalBERT-fgm^(+)^ | 90.46 | 90.85 | 90.82 | 91.04 | 90.92 | 90.82 ± 0.22 |
| **URNet-GT-fgm^(+)^** | **93.05** | **93.48** | **93.37** | **93.56** | **93.40** | **93.37 ± 0.19** |

**Supplemental Table S6.** Five-fold cross-validation results (F1 Score). The table presents the results of five-fold cross-validation for the F1 score across different methods. The mean F1 score and standard deviation (Std) for each method are also provided. The results show how the methods perform across different folds, with the best results highlighted in bold. “(+)” indicates training on the augmented dataset, while “fgm” refers to training with FGM. Standard deviation values represent the variation in performance across the different folds.

| Method | Fold 1 (%) | Fold 2 (%) | Fold 3 (%) | Fold 4 (%) | Fold 5 (%) | Mean ± Std (%) |
| --- | --- | --- | --- | --- | --- | --- |
| BERT | 82.85 | 83.00 | 83.13 | 83.28 | 82.85 | 83.02 ± 0.19 |
| BioBERT | 87.78 | 88.12 | 88.01 | 88.19 | 87.95 | 88.01 ± 0.16 |
| ClinicalBERT | 86.94 | 87.10 | 87.12 | 87.42 | 87.01 | 87.12 ± 0.18 |
| URNet-GT | 88.49 | 88.66 | 88.76 | 88.89 | 88.62 | 88.68 ± 0.15 |
| BERT^(+)^ | 84.99 | 84.91 | 85.24 | 85.33 | 85.25 | 85.14 ± 0.18 |
| BioBERT^(+)^ | 87.33 | 87.54 | 87.46 | 87.67 | 87.25 | 87.45 ± 0.17 |
| ClinicalBERT^(+)^ | 88.00 | 88.30 | 88.41 | 88.33 | 88.22 | 88.25 ± 0.16 |
| URNet-GT^(+)^ | 91.70 | 92.04 | 91.90 | 92.08 | 91.75 | 91.89 ± 0.17 |
| BERT-fgm | 86.26 | 86.46 | 86.74 | 86.60 | 86.40 | 86.49 ± 0.18 |
| BioBERT-fgm | 87.90 | 88.08 | 88.01 | 88.30 | 87.78 | 88.01 ± 0.20 |
| ClinicalBERT-fgm | 87.99 | 88.09 | 88.23 | 88.42 | 88.02 | 88.15 ± 0.18 |
| URNet-GT-fgm | 90.40 | 90.60 | 90.57 | 90.80 | 90.50 | 90.57 ± 0.15 |
| BERT-fgm^(+)^ | 86.49 | 86.90 | 86.78 | 87.00 | 86.80 | 86.79 ± 0.19 |
| BioBERT-fgm^(+)^ | 90.23 | 90.54 | 90.45 | 90.61 | 90.24 | 90.41 ± 0.17 |
| ClinicalBERT-fgm^(+)^ | 90.76 | 91.06 | 91.00 | 91.23 | 91.06 | 91.02 ± 0.17 |
| **URNet-GT-fgm^(+)^** | **93.25** | **93.60** | **93.42** | **93.65** | **93.50** | **93.48 ± 0.16** |

**Supplemental Table S7.** Five-fold cross-validation results (Accuracy). The table shows the results of five-fold cross-validation for accuracy across various models. The mean accuracy and standard deviation (Std) for each method are provided, with the best results marked in bold. “(+)” indicates training on the augmented dataset, while “fgm” refers to training with FGM. Standard deviation values represent the variability in accuracy performance across the folds.

| Department | Number |
| --- | --- |
| Orthopedics and Spinal Surgery | 217 |
| Gastrointestinal surgery | 163 |
| Department of stomatology | 150 |
| Brain surgery | 117 |
| Hepatobiliary and Pancreatic Surgery | 112 |
| Neurosurgery | 99 |
| Breast and Thyroid Surgery | 99 |
| Ophthalmology | 85 |
| Sports Medicine Department | 77 |
| Endoscopic Hepatobiliary Surgery | 73 |
| Urology Surgery | 73 |
| Hand microsurgery | 73 |
| Thoracic surgery | 67 |
| Otolaryngology | 62 |
| Cardiac surgery | 46 |
| Cardiovascular surgery | 37 |
| Gynecology | 31 |
| Baby friendly area | 23 |
| Surgical surgery | 16 |
| Gastroenterology | 11 |
| Surgical Building ICU | 10 |
| ICU Ward | 8 |
| Family Planning Department | 6 |
| Spine Surgery | 6 |
| Plastic Surgery | 4 |
| Cardiology ICU | 4 |
| Gynecological Second Ward | 4 |
| Otolaryngology Ward | 3 |
| Hand and oral surgery section | 3 |
| Hepatobiliary surgery | 2 |
| Pain clinic | 2 |
| Department of Cardiovascular Medicine | 2 |
| Intracardiac second region | 2 |
| Minimally Invasive Intervention Department | 2 |
| Intervention department | 2 |
| Rehabilitation Medicine Department | 1 |

**Supplementary Table S8.** Department statistics on the number of surgeries leading to URs from 2015 to 2021.
